# Profilin is involved in G1 to S phase progression and mitotic spindle orientation during *Leishmania donovani* cell division cycle

**DOI:** 10.1371/journal.pone.0265692

**Published:** 2022-03-22

**Authors:** Bindu Ambaru, Ganesh Muthu Gangadharan, Hosahalli S. Subramanya, Chhitar M. Gupta

**Affiliations:** 1 Institute of Bioinformatics and Applied Biotechnology, Bengaluru, Karnataka, India; 2 Manipal Academy of Higher Education, Manipal, Karnataka, India; Centre de Recherche en Biologie cellulaire de Montpellier, FRANCE

## Abstract

Profilin is a multi-ligand binding protein, which is a key regulator of actin dynamics and involved in regulating several cellular functions. It is present in all eukaryotes, including trypanosomatids such as *Leishmania*. However, not much is known about its functions in these organisms. Our earlier studies have shown that *Leishmania* parasites express a single homologue of profilin (LdPfn) that binds actin, phosphoinositides and poly- L- proline motives, and depletion of its intracellular pool to 50%of normal levels affects the cell growth and intracellular trafficking. Here, we show, employing affinity pull-down and mass spectroscopy, that LdPfn interacted with a large number of proteins, including those involved in mRNA processing and protein translation initiation, such as eIF4A1. Further, we reveal, using mRNA Seq analysis, that depletion of LdPfn in *Leishmania* cells (LdPfn^+/-^) resulted in significantly reduced expression of genes which encode proteins involved in cell cycle regulation, mRNA translation initiation, nucleosides and amino acids transport. In addition, we show that in LdPfn^+/-^ cells, cellular levels of eIF4A1 protein were significantly decreased, and during their cell division cycle, G1-to-S phase progression was delayed and orientation of mitotic spindle altered. These changes were, however, reversed to normal by episomal expression of GFP-LdPfn in LdPfn^+/-^ cells. Taken together, our results indicate that profilin is involved in regulation of G1-to-S phase progression and mitotic spindle orientation in *Leishmania* cell cycle, perhaps through its interaction with elF4A1 protein.

## Introduction

Profilin is a key regulator of actin dynamics that plays a central role in almost all vital cellular processes, including endocytosis, motility, signal transduction, metabolism, cell division, etc. [1–3]. It is a 16kDa actin-binding protein that has been recognized as one of the crucial proteins for cell survival [4]. Besides binding to actin, profilin also binds to a large array of actin-binding membrane proteins that contain poly-L-proline (PLP) motives in their structure, and through these interactions, it facilitates membrane protrusion and cell motility [5, 6]. The other major membrane ligand class of profilin is phosphoinositide lipids, and through these interactions, profilin is linked to a plethora of cellular functions [6]. Profilin is present in all eukaryotic cells, including trypanosomatid parasites, such as *Trypanosoma* and *Leishmania* [7–9]. RNAi RIT Seq data of *Trypanosoma brucei* has revealed that depletion of the intracellular pool of profilin reduces the fitness of both the procyclic and bloodstream forms, which suggests that this protein is essentially required for the survival of these parasites [10]. Similarly, complete depletion of the intracellular pool of profilin has been reported to be lethal to *Leishmania donovani* (*L*. *donovani)* promastigotes [11].

Our earlier studies have shown that *Leishmania* parasites express only a single homologue of profilin (LdPfn), which is localized to the cytoplasm, the nucleus, the kinetoplast, and the flagellum in *L*. *donovani* promastigotes [11]. LdPfn binds to monomeric actin as well as the PLP motives and phosphoinositides [11]. It has further been shown that at low concentrations, LdPfn catalyses actin polymerization, whereas, at high concentrations, it inhibits the polymerization process by sequestering actin monomers [11]. In addition, these studies have revealed that in single knockout LdPfn mutants (also called simply ‘LdPfn depleted’ cells) the cell growth was significantly retarded, and the intracellular vesicle trafficking was adversely affected [11]. As profilin is known to interact with multiple cellular ligands and through these interactions, it regulates a number of cellular functions, including cell division, gene transcription, mRNA splicing, translation and stability, cell signalling, etc [5, 6], we have now undertaken detailed proteomic and transcriptomic analyses on the wild type (LdPfn^+/+^) and LdPfn depleted (LdPfn^+/-^) *L*.*donovani* promastigotes and then validated some of the major findings drawn from these analyses by cell biological studies.

The LdPfn interactome in *Leishmania* promastigotes (LdPfn^+/+^ cells) was analysed, after affinity pull-down, by mass spectrometry, whereas the transcriptomic analysis to identify differentially expressed genes in LdPfn^+/-^ cells was carried out by high-throughput RNA sequencing (RNA-Seq) of mRNA transcripts isolated from both the LdPfn^+/+^ and LdPfn^+/-^ cells. The major findings from these studies suggested that LdPfn might be involved in regulation of the cell division cycle and mitochondrial activity in *Leishmania* promastigotes. Further studies were then carried out to confirm the role of LdPfn in *Leishmania* cell cycle, employing flowcytometry and immunofluorescence microscopy. Taken together, the results of these studies revealed that LdPfn is involved in regulation of the G1-to-S phase progression and mitotic spindle orientation in *Leishmania* cell cycle, plausibly through its interaction with eukaryotic translation initiation factor 4A1(eIF4A1).

## Materials and methods

### Leishmania culture

High glucose Dulbecco’s modified Eagle’s medium (DMEM) (Life Technologies, Thermo Fisher Scientific) supplemented with 10% heat-inactivated foetal bovine serum (MP chemicals) and 4mg ml^-1^ gentamicin (MP chemicals) was used to maintain *Leishmania donovani* (Strain: MHOM/IN/80/DD8; ATCC Cat.No.50212) cells. Heterozygous LdPfn mutants (LdPfn^+/-^) were prepared as described earlier [11] and maintained in high-glucose DMEM supplemented with 10% heat-inactivated foetal bovine serum and 100μg ml^-1^ hygromycin B (Invitrogen) at 25°C. The mutants ectopically complemented with LdPfn gene (LdPfn^+/-comp^) were maintained in the same medium with dual antibiotic pressure of G418 and hygromycin B at 100μg ml^-1^ concentration each. Construction of clones, transfection in *Leishmania* promastigotes, and validation for protein expression was recorded as described earlier [11].

### Pull-down assay and mass spectrometry

Full-Length profilin was cloned and expressed with GST-tag (GST-LdPfn), as described earlier [11]. For the pull-down assay, purified recombinant GST-LdPfn or GST alone was incubated with 100μL of Glutathione-Sepharose affinity beads for 2 hours. The unbound proteins were removed by centrifugation at 1000 x g for 10 minutes at 4°C and the recombinant proteins associated with the beads were incubated with 2mg protein of clear lysate of *L*. *donovani* promastigotes (clear supernatant obtained after sonication and centrifugation) overnight at 4°C. The next day, unbound proteins were removed, and the beads were washed five times with 1x PBS. Finally, the bound proteins were eluted by boiling the beads at 96°C for 5 min in 50ul of SDS- poly acrylamide gel electrophoresis (SDS-PAGE) loading buffer (250mM Tris, 10% SDS, 0.5% bromophenol blue, 50% glycerol, and 500mM 2-mercaptoethanol). Eluates were run on 12% polyacrylamide gel. Three biological replicates of each of GST and GST-LdPfn pull-down samples were stained with silver nitrate, the gel was rinsed thrice with double distilled water; the respective lanes were excised, kept in labelled Eppendorf tubes submerged with double distilled water, and submitted at the mass spectrometry facility (Institute for Stem Cell Regeneration and Medicine, NCBS-TIFR Campus, Bellary Road, Bangalore Karnataka, India) for analysis, using LC-MS (Orbitrap Fusion™ Tribrid™ Mass Spectrometer). Only the protein hits with a score cut off 25, significance threshold p<0.05 were considered. The data thus-obtained were used for searching the www.tritrypdb.org database (version 51), using the *Leishmania donovani* (BPK282A1) strain as reference. Data has been deposited at PRIDE–ProteomeXchange Consortium [12] and can be accessed with the identifier: PXD026036. Among the three biological replicates, the proteins present in all three replicates or at least two replicates of GST-LdPfn, but not present in any of the replicates of GST control, were taken into consideration.

### Western blotting

*Leishmania* cell lysates were prepared by re-suspending and boiling the cell pellets for 5 minutes in an SDS-PAGE sample buffer. Samples were resolved on SDS-polyacrylamide gel by electrophoresis and electro-blotted onto nitrocellulose membrane (0.45μm, Synergy scientific services) in Tris-glycine buffer (pH 8.3) at 80V for 3 hours. The membrane was treated with 5% skimmed milk to block the nonspecific sites, and then probed with rabbit anti-LdPfn antibodies [11] (1:100 dilution) or mouse anti-β tubulin monoclonal antibodies (Sigma, cat.No. T7816) (1:5000) or rabbit anti-eIF4A.1 antibodies (Cell Signalling Technology, cat No. 2490) (1:100) for overnight at 4°C. The membrane was washed 5 times with Tris-buffered saline (pH 7.5) containing 0.05% (v/v) Tween 20, then incubated with HRP conjugated anti-rabbit IgG antibody (Invitrogen, cat no. A16074) or HRP conjugated anti-mouse IgG antibody (Invitrogen, cat no.62-6820) for 2 hours and developed with ECL (Bio-Rad, Clarity) and imaging software (Syngene, G-box).

### Total RNA isolation and library construction

Total RNA from three independent biological replicates was isolated from LdPfn^+/+^ (control) and LdPfn^+/-^ promastigotes using TRIzol reagent (Ambion, Life Technologies), according to the manufacturer’s instructions. RNA samples were treated with DNase I (2μg) and the RNA concentration was determined, using a spectrophotometer at A260/280 (Nanodrop ND1000, Thermo Scientific, USA). In addition, the RNA integrity was evaluated using TapeStation (Agilent) and Qubit (Invitrogen). Using NEBNextUltra^TM^ II RNA Library Prep Kit for Illumina, Poly (A) mRNA magnetic isolation was performed. Library preparations were carried out, using the NEBNextUltra^TM^II RNA Library Prep kit (Illumina), according to the manufacturer’s instructions.

### RNA-Seq and data analysis

Paired-end reads (2 x 150 bp) were obtained using the Illumina HiSeq 2500 platform at the Bio-IT centre at the Institute of Bioinformatics and Applied Biotechnology. Raw data were generated for each of the libraries from 6 samples. The quality of the produced data was analysed using FastQC by Phred quality score. Reads with Phred quality scores lower than 20 were discarded. Reads were aligned to the *L*, *donovani* (BPK282A1) genomic data obtained from TriTrypDB version 51 (www.tritrypdb.org), using Bowtie2 (-x option) [13, 14]. All analyses were carried out using the Tophat pipeline with the following versions: Tophatv2.1.1, Bowtie2 v2.3.51 [15]. Tophat is a fast splice junction mapper for RNA-Seq reads. It aligns RNA-Seq reads to genomes, using the ultra-high throughput short read aligner Bowtie and then analyses the mapping results to give the transcript counts. The gene expression level values were calculated from the transcript counts. Gffread tool [16] was used to convert gff files to gtf files for read-count calculation using HTSeq. The HTSeq version 0.12.4 (htseq-count -f option) was used to count the number of reads aligned to protein-coding genes. HTSeq [17, 18] is a Python package that gives the infrastructure to processed data from high-throughput sequencing assays. HTSeq calculates the number of mapped reads to each gene. DeSeq tool was used for differential gene expression analysis between samples in protein-coding genes. DeSeq [18, 19] is an R package to measure variance-mean dependence in count data from high-throughput RNA-Seq assays and test for differential expression depending on a model applying the negative binomial distribution. Differentially expressed (DE) genes were classified as genes with a Benjamini-Hochberg multiple testing p-value of <0.05. Principal component analysis plot (PCA plot), clustered heat map, and volcano plots were created using base R plot PCA function (or base R prcomp function), gplotsheatmap.2 functions and ggplot function of gglplot2 package respectively. Functional annotation was performed using GO (gene ontology) and the Kyoto Encyclopaedia of Genes and Genomes (KEGG) and Metabolic Pathways from all Domains of Life (MetaCyc) using the *L*.*donovani* (BPK282A1) GO annotations provided in TriTrypDB.

### Real time quantitative polymerase chain reaction (RT-qPCR) validation assays

DNase-treated RNA (2μg) was reverse transcribed with MMLV reverse transcriptase (NEB). Equal amounts of cDNA were assessed in triplicate in a total volume of 10ul containing SYBR Green (Kapa Biosystems) and the primers used were given in S1 Table. The mixture was incubated at 95°C for 20 sec, 55°C for 30 sec, and 72°C for 1 sec for 40 cycles. Negative controls were included in the RT-qPCR assays to detect DNA contamination in RNA samples. The fold-change in expression of up and down regulated genes was determined, using RT-qPCR. Reactions were carried out on a fast RT-qPCR system (Applied Biosystems). The results were quantified by the delta-delta CT method with actin as a reference gene and β-tubulin as an endogenous control to normalize each sample. The specificity of the reaction was verified by melt curve analysis. All the experiments were conducted at least three times and the results were expressed as mean ± SEM of three independent experiments.

### Immunofluorescence microscopy

Cells were washed with PBS and were adhered to poly-L-lysine coated glass coverslips for 15 min. They were then fixed with 2% paraformaldehyde (PFA) for 30 minutes at room temperature and washed thoroughly with PBS containing 0.5% glycine (w/v). The washed cells were permeabilized, using 0.5% (v/v) Triton X-100 for 15 minutes, and washed with PBS-glycine. The washed cells were blocked with 3% bovine serum albumin in PBS for 2 hours at 25°C and labelled with anti-LdPfn antibodies [11] (1:100) and or anti-tubulin antibodies (Santacruz biotechnologies cat no. sc-5286 and Sigma cat.no. T7816) (1: 500) overnight at 4°C. The labelled cells were washed with 0.5% bovine serum albumin in PBS to remove non-specifically bound antibodies and again labelled with Alexa Fluor 568 –conjugated goat anti-mouse IgG (Thermo Fisher Scientific, cat no. A21043) or Alexa Fluor 488– conjugated goat anti-rabbit IgG (Thermo Fisher Scientific, cat no. A32731) or Fluor 488– conjugated goat anti-mouse IgG (Thermo Fisher Scientific, cat no.A32723) or Alexa Fluor 568–conjugated goat anti-rabbit IgG (Thermo Fisher Scientific, cat no.A21069) secondary antibodies depending on the experiment. The coverslips were mounted using prolong diamond anti-fade mounting media containing 4,6-diamidino-2-phenylindole (DAPI, Invitrogen). To analyse the nuclear division patterns, the coverslips were treated with RNase (5mg/ml) followed by labelling with propidium iodide (PI). Images were captured on Nikon laser scanning confocal microscope c2 using a 100X1.4 NA (oil) plan apochromatic lens.

### Cell cycle analysis

For cell cycle analysis, *Leishmania* cultures (5 x 10^7^cells) were synchronized by incubating them with 200 mg ml^-1^ of N-hydroxyurea (HU) (Sigma) overnight (12–14 hours). The cells were washed and then re-suspended in DMEM media containing 10% FCS without HU. Aliquots of cell suspension were drawn at 0 hour to 10 hours at regular time intervals of 2 hours. The cells were washed with cold PBS and resuspended in 50μl of PBS. The cell suspension was mixed with 150μl of fixative solution (1% Triton X-100, 40mM citric acid, 20mM sodium phosphate, 200mM sucrose) and incubated at 25°C for 5 minutes. After incubation, 350μl of diluent buffer (125mM MgCl_2_ in PBS) was added to it and stored at 4°C until further use. Before the cell cycle analysis, the fixed cell suspension was treated with 50μg RNase (5mg ml^-1^in 0.2M sodium phosphate buffer, pH7.0) for 2 hours at 37°C and then incubated with 50μg PI (5mg ml^-1^ in 1.12% sodium citrate) for 30 minutes at 25°C. The samples were analysed in Gallios flow cytometer (Beckman coulter) and proportions of the G1, S, and G2M populations were determined using ModFit LT software (Verity Software House, Topsham, ME, USA).

### Bromo deoxy uridine incorporation assay

*Leishmania* cells either synchronized with 200 mg ml^-1^ of HU for 12–14 hours or mid-log phase asynchronous culture was treated with 5-Bromo-2’-deoxyuridine (BrdU, Sigma) at a final concentration of 30μM. At each time point, cells were incubated strictly for 1hour with BrdU at 25°C, washed with PBS, and fixed with 4% PFA until the next day. Cells were washed and treated with 4N HCl containing 0.5% (v/v) Triton X-100 for 30 minutes (in situ DNA denaturation) to allow access of anti-BrdU antibody (Sigma, cat no: B2531) to BrdU substituted groups in the newly synthesized DNA. Cells were washed with 0.1M sodium tetraborate to quench residual HCl followed by washing once with 1% BSA in PBS, pH 7.4. The cells were resuspended in 1% BSA in PBS containing 0.5% Tween 20 and treated with primary anti-BrdU antibody (1:1000) for 2.5 hours at 25°C. The cells were again washed and re-suspended in 1% BSA in PBS containing 0.5% Tween 20 and treated with Alexa Fluor 488 –conjugated goat anti-mouse IgG secondary antibodies (1:500; Life technologies, thermos fisher scientific, cat no: A-10680) for 1 hour at 25°C. The cells were washed and re-suspended in PBS with 10μg ml^-1^ RNase and 2μg of PI. The incorporation of BrdU was analysed in Gallios flow cytometer (Beckman coulter) and the percentage of cells labelled with BrdU was determined using WinList software (Verity Software House, Topsham, ME, USA).

### Statistical analysis

All the experiments were conducted at least three times and the results were expressed as the standard error of the mean (mean ± SEM) of three experiments. The data were statistically analysed by ANOVA test with replication. A p-value of <0.05 was considered significant.

## Results

### *Leishmania* profilin interacts with a large number of cellular ligands, including elF4A1

**Fig 1:**
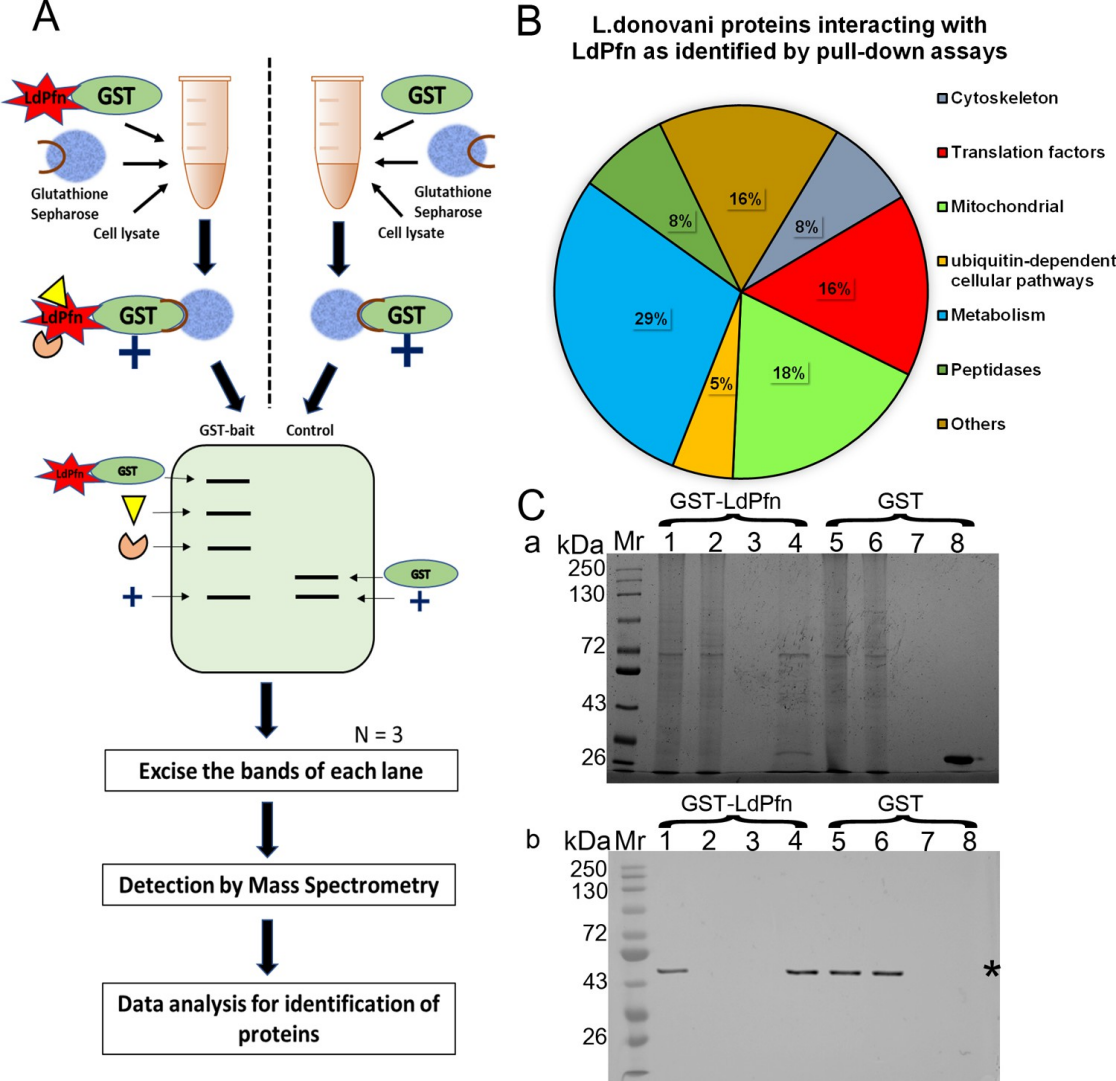
Pull-down assay, mass spectrometry analysis, and validation with western blot. **(A)** Schematic representation of the experimental design. **(B)** Pie chart showing the total number of GST-profilin ligands (38 proteins) grouped according to their deduced function or cellular location. The percent of proteins in each group has been labelled as Cytoskeleton (8%), Translation factors (16%), Mitochondrial (18%), Ubiquitin-dependent cellular pathways (5%), Metabolism (29%), Peptidases (8%), and Others (16%). **(C)** Validation of Proteomics results with western blot analysis. **(a)** Silver-stained 12% polyacrylamide gel. Mr, molecular weight markers; lanes 1–4 correspond to GST-*Leishmania* profilin (GST-LdPfn) pulldown: lane 1, Input lysate; lane 2, unbound fraction; lane 3, wash fraction; lane 4, GST-LdPfn pulldown eluate; lanes 5–8 correspond to GST- alone pulldown: lane 5, Input lysate; lane 6, unbound fraction; lane 7, wash fraction; lane 8, GST-alone pulldown eluate. **(b)** Western blot of lanes 1 to 8 from **‘a**’ with anti-eIF4A-1 antibodies. Asterisk marks the band corresponding to eIF4A-1 protein (45.3 kDa).

To map the ligand binding profile of LdPfn, we performed a pull-down assay in lysates of mid-log phase *Leishmania* promastigotes, using glutathione S-transferase (GST)-tagged LdPfn [11] and GST protein alone (negative control), as a bait, as described in ‘Materials and Methods’. LdPfn bound to its ligands was isolated by using glutathione-Sepharose affinity beads. The beads were washed thoroughly to remove unbound proteins and the bound proteins were eluted by boiling the beads in SDS-polyacrylamide gel electrophoresis (PAGE) sample loading buffer. The eluates from three independent pull-down experiments of both GST-LdPfn and GST-alone were resolved on SDS-PAGE. The gels were silver-stained, excised, and subjected to trypsin digestion followed by liquid chromatography-mass spectrometry (LC-MS) analysis (Fig 1A). The proteins present in all three replicates (4 proteins) and those present in at least two replicates (34 proteins) of GST-LdPfn pull-down, but not present in any one of the GST-alone pull-down controls are listed in Table 1. A summary of each protein hit is given in S2 Table. The LdPfn ligands thus detected were classified according to their deduced function or cell location, and the percent of proteins representing each group are shown in Fig 1B.

**Table 1:** *L*.*donovani* proteins identified in three pull-down assays with GST-profilin using LC-MS.

#	TriTrypdb ID	Protein Description	Deduced function/location
** Cytoskeleton**			
1	**LDBPK_320550.1**	Profilin*	Actin sequestering and polymerization [11]
2	**LDBPK_041250.1**	Actin*	Microfilament [66]
3	LdBPK_362370.1	Gamma-tubulin complex component 3-like protein	Microtubule [20]
** Translation factors**			
4	LdBPK_010790.1	Eukaryotic initiation factor 4A-1	Translation initiation factor [23, 24]
5	LDBPK_170010.1	Eukaryotic translation initiation factor 3 subunit a	Translation initiation factor [67]
6	**LdBPK_360210.1**	Elongation factor 2*	TH1 stimulatory protein [68]
7	LDBPK_353150.1	ATP-dependent RNA helicase, putative	pre mRNA splicing and spliceosome assembly [23]
8	LdBPK_322350.1	U5 small nuclear ribonucleoprotein component, putative	pre mRNA splicing and spliceosome assembly [69]
9	LdBPK_365870.1	Isoleucyl-tRNA synthetase, putative	Catalyzes the specific attachment of an amino acid to its cognate tRNA [70]
** Mitochondrial activity**			
10	**LDBPK_020430.1**	Mitochondrial outer membrane protein porin, putative*	Metabolite transporter [22]
11	LDBPK_241700.1	Succinate dehydrogenase [ubiquinone] flavoprotein subunit, mitochondrial	SDH complex is located on the inner membrane of the mitochondria and participates in both the citric acid cycle and the respiratory chain [71]
12	LdBPK_362790.1	Dihydrolipoamide acetyltransferase precursor, putative	Component of mitochondrial pyruvate dehydrogenase complex [71]
13	LDBPK_281310.1	Luminal binding protein 1 (BiP), putative	ER binding, protein folding [72]
14	LdBPK_180510.1	Aconitase, putative	Enzyme in redox reaction [73]
15	LdBPK_271770.1	Trypanothione synthetase	Enzyme in redox reaction [74]
16	LDBPK_090820.1	Oligopeptidase b (OBP)	Enzyme in redox reaction [75]
**Ubiquitin-dependent cellular pathways **			
17	LDBPK_361420.1	Valosin-containing protein, putative	Ubiquitin-dependent cellular pathways, essential for intracellular development of *Leishmania* [21]
18	LDBPK_343890.1	Ubiquitin hydrolase, putative	Function in protein deubiquitination (DUB18) [76]
** Metabolism and signaling**			
19	LdBPK_362720.1	Membrane-bound acid phosphatase 2, putative	Integral component of membrane [77]
20	LDBPK_362480.1	Glyceraldehyde-3- phosphate dehydrogenase, cytosolic	Part of the glycolysis pathway [78]
21	LDBPK_313250.1	Phosphatidylethanolamine-methyltransferase-like protein	Methylation enzyme-2 [79]
22	LDBPK_242150.1	Transketolase	Metabolism [80]
23	LDBPK_290120.1	Proteasome regulatory non-ATPase subunit, putative	Proteasome non-ATPase regulatory subunit [81]
24	LDBPK_281850.1	Proteasome regulatory non-ATPase subunit 2, putative	Proteasome non-ATPase regulatory subunit [81]
25	LdBPK_020680.1	ATP-dependent Clp protease subunit, heat shock protein 78 (HSP78), putative	ATP-dependent Clp protease subunit, heat shock protein 78 (HSP78) [82]
26	LDBPK_181350.1	Heat shock protein, putative	Heat shock protein show increased phosphorylation, indicating a role in stage-specific signal transduction [82]
27	LDBPK_140700.1	Fatty acid elongase, putative	An enzyme that catalyzes fatty acid synthesis [83]
28	LdBPK_030220.1	Long-chain fatty Acyl CoA synthetase, putative	An enzyme that catalyzes fatty acid synthesis [83]
29	LDBPK_140670.1	Fatty acid elongase, putative	An enzyme that catalyzes fatty acid synthesis [83]
**Peptidases**			
30	LdBPK_040430.1	Calpain-like cysteine peptidase, putative	Peptidase [84]
31	LdBPK_110640.1	Metallo-peptidase, Clan MF, Family M17 (fragment)	Peptidase [85]
32	LDBPK_050960.1	Dipeptidyl-peptidase III, putative	Proteolysis [86]
** Others**			
33	LdBPK_161510.1	Paraflagellar rod protein 2C	Motility [87]
34	LDBPK_131250.1	AAA domain/Dpy-30 motif-containing protein, putative	Protein folding
35	LdBPK_100510.1	GP63, leishmanolysin (fragment)	Leishmanolysin is the predominant protein surface antigen of promastigotes and is assumed to have a key role during infection [88]
36	LDBPK_332400.1	Hypothetical protein, conserved	Unknown
37	LdBPK_323200.1	Hypothetical protein, conserved	Unknown
38	LdBPK_161280.1	Hypothetical protein, conserved	Unknown

Proteins identified in the three GST-profilin pull-down assays are shown in bold with an asterisk, while others are proteins identified in at least two GST-profilin pull-down assays. Only the proteins that were not detected in any of the GST pull-down controls were considered. The protein IDs were given according to *L*. *donovani* BPK282A1 strain as downloaded from the TriTrypDB (version 51) database (www.tritrypdb.org).

Some of the LdPfn ligands, such as actin (LdBPK_041250.1), valosin-containing protein (LDBPK_361420.1), mitochondrial outer membrane protein porin (LDBPK_020430.1), and eIF4A1 (LdBPK_010790.1), have earlier been identified as the potential ligands also of the *T*. *cruzi* profilin [8]. Interestingly, unlike *T*. *cruzi* [8], we have also identified *gamma-*tubulin complex component 3-like protein (LdBPK_362370.1), a key component of the microtubule-organizing centre [20], as one of the potential ligands of LdPfn. While valosin-containing protein VCP/p97 has been shown to be essential for the intracellular development of *Leishmania* [21], *T*. *brucei* porin (Tb927.2.2510) has been reported to be the main metabolite channel in the mitochondrial outer membrane and is required to support efficient oxidative phosphorylation [22]. Further, the eukaryotic initiation factor 4A1 (eIF4A1) is abundantly present in *Leishmania* cytoplasm [23], and this protein has been suggested to be the main translation initiation factor involved in protein synthesis in *T*. *brucei* [24]. To further confirm the presence of this protein in the LdPfn interactome, we analysed the pull-down eluates from GST-LdPfn and GST-alone by western blotting, using anti-eIF4A1 antibodies. Results given in Fig 1C and S1 Fig show that eIF4A1 (45.3kDa) was present in the input lysates used in both the GST-LdPfn and GST-alone pull-down assays and also in the GST-LdPfn pull-down eluates and pass-through fraction from the GST-alone pull-down assay, but it was completely absent in the pass-through fraction from GST-LdPfn pull-down and GST-alone eluate. These results strongly indicate that LdPfn interacts with multiple cellular ligands, a majority of which constitute the proteins that may play an important role in regulating mRNA processing, translation initiation, cell metabolism and mitochondrial functions.

As LdPfn appeared to interact with proteins involved in mRNA processing (LDBPK_353150.1 and LdBPK_322350.1) and protein translation initiation (elF4A1), we performed transcriptomic analysis of mRNA isolated from mid-log phase LdPfn^+/+^ and LdPfn^+/-^ cells, employing high throughput RNA sequencing technique (RNA-Seq), to identify differentially expressed genes in LdPfn^+/-^ cells.

### Transcriptomic data analysis revealed that the genes involved in DNA transcription, mRNA translation, cell cycle regulation, membrane transport, and mitochondrial activity were differentially expressed in LdPfn depleted cells

*L*. *donovani* (BPK282A1) contains 36 chromosomes and has a haploid genome size of 32.44 Mb, which encodes a total of 8135 genes (8023 protein-coding genes and 112 non-protein-coding genes) [25]. These parasites display a unique way of controlling their gene expression in that the mRNAs are made from polycistronic precursors by SL-*trans* splicing and polyadenylation [26–28]. Many protein-coding genes of unrelated functions are arrayed in long clusters on the same DNA strand. Intergenic regions of polycistronic pre-mRNAs are co-transcriptionally processed by two reactions: polyadenylation of the upstream gene and trans-splicing of the capped mini exon to the downstream gene [29], thus generating monocistronic units ready for degradation or translation. It is presumed that all polycistronic precursor RNAs are transcribed approximately at the same rate. As a consequence, the regulation of gene expression occurs most entirely post-transcriptionally [30]. To identify differentially expressed genes in profilin depleted *Leishmania* promastigotes, we analysed the transcriptomes of both the mid-log phase LdPfn^+/+^ and LdPfn^+/-^ *Leishmania* promastigotes, using high-throughput RNA sequencing technique (RNA-Seq). Total RNA from three independent biological replicates of each LdPfn^+/+^ and LdPfn^+/-^ promastigotes was extracted and after library preparation, RNA sequencing was carried out on Illumina HiSeq 2500. The RNA-Seq datasets generated in the analysis with each sample have at least 30 million read pairs (S2 Fig and S3 Table). Principal Component Analysis (PCA) was used to analyse the relationship between the samples (S3 Fig), showing a clear separation between LdPfn^+/+^ and LdPfn^+/-^ samples. RNA-Seq data were aligned to the *L*. *donovani* genome (*L*. *donovani* BPK282A1, NCBI taxon ID: 981087). 8135 transcripts were identified in the data set, which correlated with the data reported earlier [25].

**Fig 2:**
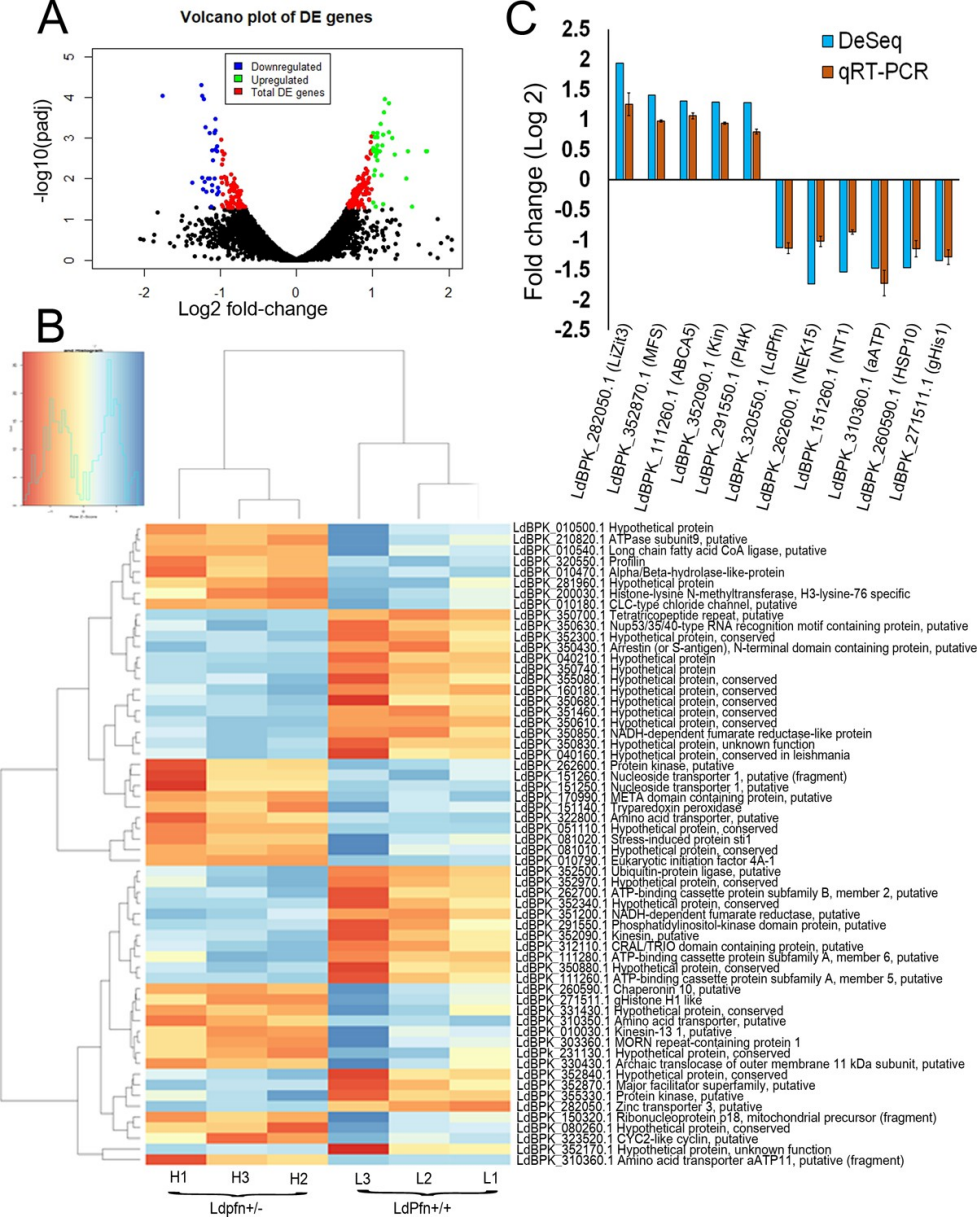
Differentially expressed genes. **(A)** Volcano plot of differentially expressed genes. The x-axis represents the log 2-fold change and y-axis represent -log10 (adj P-value). Green dots represent up-regulated genes, blue dots represent down-regulated genes and red dots represent other differentially expressed genes. **(B)** Heatmap of top 30 up-regulated and down-regulated genes following hierarchical clustering analysis. L1, L2, L3 are the three replicates of controls (LdPfn+/+), and H1, H2, H3 are the three replicates of single knockout samples (LdPfn+/-). The horizontal axis represents the samples, and the vertical axis represents the differentially expressed genes (DEGs). Red indicates down-regulated genes and blue indicates up- regulated genes in LdPfn+/- cells. **(C)** Comparative analysis of the relative expression levels of selected transcripts determined by RNA-Seq and validated by RT-qPCR. Based on the RNA-Seq (DeSeq) analysis, five up-regulated transcripts: LdBPK_282050.1.1 (Zinc transporter 3, putative), LdBPK_352870.1.1 (major facilitator superfamily, putative), LdBPK_111260.1.1 (ATP-binding cassette protein subfamily A, member 5, putative), LdBPK_352090.1.1 (kinesin, putative), LdBPK_291550.1.1 (phosphatidylinositol-kinase domain protein. putative) and five down-regulated transcripts: LdBPK_262600.1.1 (protein kinase, putative), LdBPK_151260.1.1 (nucleoside transporter 1, putative (fragment)), LdBPK_310360.1.1 (amino acid transporter aATP11, putative (fragment)), LdBPK_260590.1.1 (Chaperonin 10, putative), LdBPK_271511.1.1 (g histone H1 like) and LdBPK_320550.1.1 (profilin) were selected for validation by RT-qPCR. The RT-qPCR experiments were conducted at least three times and the results are expressed as mean ± SEM.

RNA-Seq data revealed that 254 genes were differentially expressed (DE) having an adjusted p-value <0.05. Based on the DE genes, volcano plots were generated (Fig 2A), showing distribution of the transcripts by comparing the fold change in the expression (log_2_) of each group with the corresponding adjusted p-value (-log_10_). Among these DE genes, 112 genes (~44%) were hypothetical with unknown functions. The complete list of differentially expressed (DE) genes with known functions either upregulated or downregulated and those with unknown functions along with their respective transcript IDs and log2 fold change are listed in S4 Table. Further, a clustered heat map was generated with the top 30 up and top 30 down-regulated genes to evaluate the reproducibility of the biological replicates (Fig 2B). The data obtained from RNA-Seq was validated by RT-qPCR of 11 genes, 5 up-regulated and 6 down-regulated including profilin transcript (Fig 2C). Results of the RT-qPCR analysis showed a strong correlation with the RNA-Seq data, thus validating the RNA-Seq results (Fig 2C).

**Fig 3:**
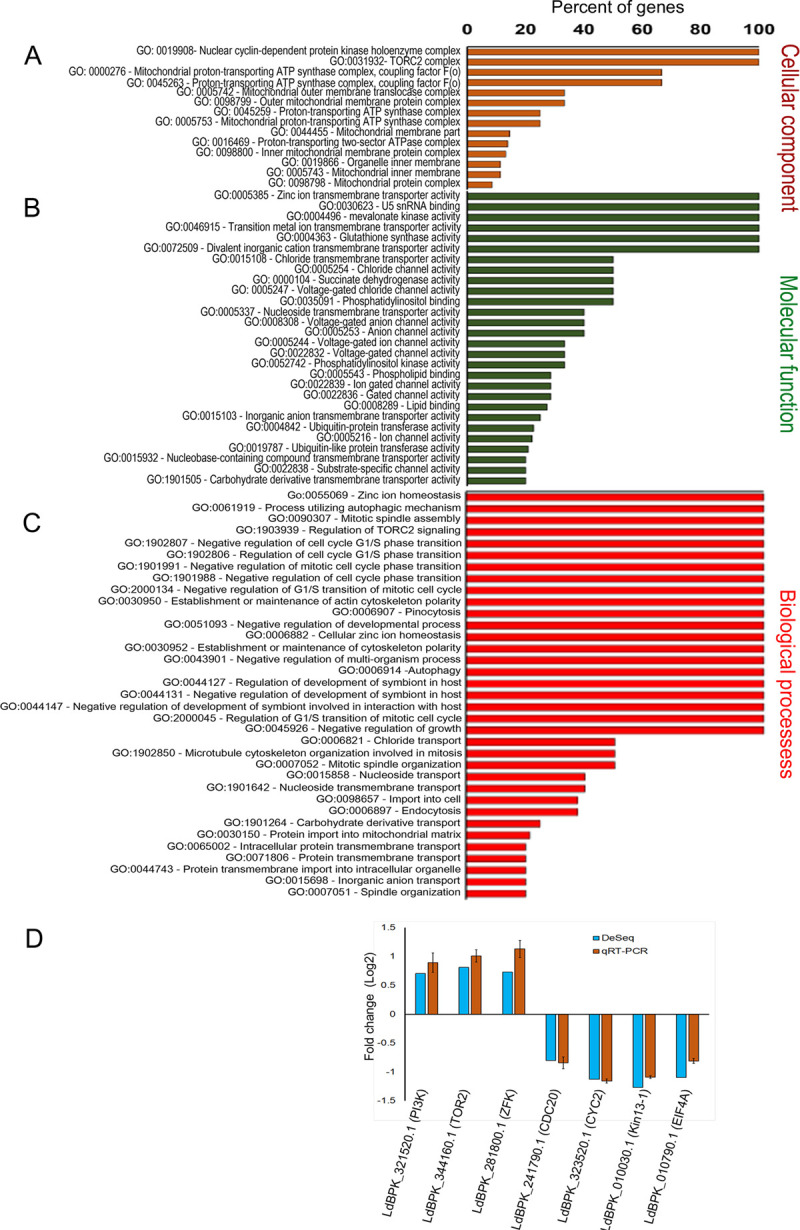
Gene Ontology annotation for all differentially expressed genes and their validation by RT-qPCR. The vertical axis represents the Gene Ontology (GO) categories, and the horizontal axis represents the percentage of significant genes in that particular GO category. **(A)** The GO annotations of cellular component; **(B)** The GO annotations of molecular function; **(C)** The GO annotations of biological processes. The GO terms of p-value <0.05 were considered; **(D)** Comparative analysis of the relative expression levels of selected transcripts determined by RNA-Seq and by RT-qPCR. The transcripts corresponding to the genes of interest LdBPK_321520.1.1 (phosphatidylinositol 3-related kinase, putative), LdBPK_344160.1.1 (Phosphatidylinositol 3-kinase tor2), LdBPK_281800.1.1 (differentiation inhibitory kinase, putative), LdBPK_241790.1.1 (cell division cycle protein 20), LdBPK_323520.1.1 (CYC2-like cyclin, putative), LdBPK_010030.1.1 (Kinesin-13-1, putative), LdBPK_010790.1.1 (Eukaryotic initiation factor 4A-1) were validated by RT-qPCR. The RT-qPCR experiments were conducted at least three times and the results were expressed as mean ± SEM.

To evaluate the probable effects of differentially expressed genes in LdPfn^+/-^ cells, we performed gene ontology (GO) analysis of 254 DEGs to identify the cellular component, molecular functions, and biological processes enriched due to profilin depletion. Fig 3A shows that the transcripts encoding proteins were mainly localized to the nuclear component, mitochondrial complex, and TORC2 complex. The molecular functions (Fig 3B) enriched by these transcripts were mainly predicted to include the transmembrane transporter activity, phosphatidylinositol binding, kinase activities, and ubiquitin-protein transferase activity. Biological processes that were enriched due to depletion in the LdPfn levels are shown in Fig 3C. From RNA-Seq data, we identified some of the genes enriched in GO biological processes and then validated their mRNA expressions by RT-qPCR. Fig 3D shows that the expressions of LdBPK_321520.1.1 (phosphatidylinositol 3-related kinase, putative), LdBPK_344160.1.1 (Phosphatidylinositol 3-kinase tor2), LdBPK_281800.1.1 (differentiation inhibitory kinase, putative), LdBPK_241790.1.1 (cell division cycle protein 20), LdBPK_323520.1.1 (CYC2-like cyclin, putative), LdPBK_010030.1.1 (Kinesin-13 1, putative), LdBPK_010790.1.1 (Eukaryotic translation initiation factor 4A1) genes strongly correlated with the RNA-Seq data. Major findings from the RNA-Seq data (with log 2-fold change, cut off above +0.8 for upregulated and below -0.8 for down regulated genes) were grouped based on their cellular activities (Table 2) that could have been affected due to LdPfn depletion. Results given in Table 2 clearly indicate that expression of genes that are involved in regulation of mitochondrial activity (such as ATOM14, ATOM11), cell division (such as CYC2-like cyclin, cell division cycle protein 20 (CDC20) and kinesin 13.1), DNA transcription (such as histone-lysine N-methyltransferase), mRNA translation (such as eIF4A1), and amino acids and nucleosides transport was significantly reduced in LdPfn depleted cells.

**Table 2:** List of differentially expressed genes.

No	Transcript ID	Log 2-fold change	Product description	Deduced function in trypanosomatids
**Phosphoinositide metabolism**					
1	LdBPK_291550.1.1	1.280818339	Phosphatidylinositol-kinase domain protein, putative	1-phosphatidylinositol 4-kinase activity [89]
2	LdBPK_352470.1.1	0.886534182	Autophagy-related protein 24	Autophagy-related protein 24 (ATG24) [90]
3	LdBPK_350560.1.1	0.87626063	Phosphatidylinositol-4-phosphate 5-kinase-like, putative	Phosphoinositide-binding protein [89]
4	LdBPK_350040.1.1	0.839630257	Phosphatidylinositol-specific phospholipase-like protein	Phosphoinositide-binding protein (PI-PLC) [91]
5	LdBPK_344160.1.1	0.813759977	Phosphatidylinositol 3-kinase tor2	TOR2 is required for signalling organization of actin cytoskeleton during the cell cycle [92]
**Signalling activity**					
6	LdBPK_355330.1.1	1.023944623	Protein kinase, putative	Protein kinase activity and protein phosphorylation [93]
7	LdBPK_040420.1.1	0.93559282	Serine/threonine protein kinase-like protein	Protein kinase activity and protein phosphorylation [93]
8	LdBPK_352370.1.1	0.905411272	Protein kinase, putative	Protein kinase activity and protein phosphorylation [93]
9	LdBPK_303090.1.1	0.812777426	Protein kinase-like protein	Protein kinase activity and protein phosphorylation [93]
10	LdBPK_351030.1.1	0.821820017	Casein kinase, putative	Plays an important role in parasite survival and virulence [94]
11	LdBPK_161340.1.1	0.804120633	Diacylglycerol kinase, putative	Phosphorylation of diacylglycerol (DAG), converting it into phosphatidic acid (PA). Involved in lipid signalling in trypanosomatids [95]
**Transport activity**					
12	LdBPK_282050.1.1	1.941029466	Zinc transporter 3, putative	*Li*ZIP3 functions as a zinc importer in *L*. *infantum* [96]
13	LdBPK_352870.1.1	1.409207886	major facilitator superfamily, putative	Transmembrane transporter [97]
14	LdBPK_111260.1.1	1.306173522	ATP-binding cassette protein subfamily A, member 5, putative	Transporter involved in vesicular trafficking [98]
15	LdBPK_262700.1.1	1.103652155	ATP-binding cassette protein subfamily B, member 2, putative	Transporter [98]
16	LdBPK_111280.1.1	1.013600698	ATP-binding cassette protein subfamily A, member 6, putative	Transporter involved in vesicular trafficking [98]
17	LdBPK_340690.1.1	0.98628944	ATP-binding cassette protein subfamily C, member 8, putative	ABCC8 acts as intracellular transporter associated with resistance to antimonialsSb(III) [98]
18	LdBPK_100370.1.1	0.946001912	folate/biopterin transporter, putative	Transporter [99]
19	LdBPK_151260.1.1	-1.534202851	nucleoside transporter 1, putative (fragment)	Translocation of nucleosides [43, 44]
20	LdBPK_310360.1.1	-1.475208384	amino acid transporter aATP11, putative (fragment)	Amino acid transporter [42]
21	LdBPK_151250.1.1	-1.249810093	nucleoside transporter 1, putative	Translocation of nucleosides [43, 44]
22	LdBPK_322800.1.1	-1.233233795	amino acid transporter, putative	Amino acid transporter [42]
23	LdBPK_010180.1.1	-1.230763978	CLC-type chloride channel, putative	Transporter [100]
24	LdBPK_310350.1.1	-1.033934659	amino acid transporter, putative	Amino acid transporter [42]
25	LdBPK_151230.1.1	-1.000062439	nucleoside transporter 1, putative	Translocation of nucleosides [43, 44]
**Mitochondrial activity**					
26	LdBPK_260590.1.1	-1.466015198	Chaperonin 10, putative	Protein folding [101]
27	LdBPK_330430.1.1	-1.07586746	archaic translocase of outer membrane 11 kDa subunit, putative	Mitochondrial tRNA import [59]
28	LdBPK_210820.1.1	-1.030668798	ATPase subunit 9, putative	Subunit of mitochondrial ATPase complex involved in H+ transport [102]
29	LdBPK_240960.1.1	-0.929674384	Archaic Translocase of outer membrane 14 kDa subunit, putative	Protein import and tRNA import into mitochondria [59]
30	LdBPK_240640.1.1	-0.848551581	ATPase subunit 9, putative	Subunit of mitochondrial ATPase complex involved in H+ transport [102]
31	LdBPK_260610.1.1	-0.845823341	10 kDa heat shock protein, putative	The Hsp60/10 complex is believed to be responsible for accelerating the folding of polypeptides imported into mitochondria, as well as reactivation of denatured proteins, and diminishing aggregation of non-native polypeptides and partially unfolded kinetically trapped intermediates [82]
32	LdBPK_251890.1.1	-0.82565037	cytochrome c oxidase assembly protein, putative	Involved in mitochondrial electron transport [103]
33	LdBPK_070910.1.1	-0.800148385	flavoprotein subunit-like protein	Involved in the mitochondrial electron transport chain and is responsible for transferring electrons from succinate to ubiquinone (coenzyme Q) [104]
34	LdBPK_350850.1.1	1.193560347	NADH-dependent fumarate reductase-like protein	Mitochondria [105]
35	LdBPK_351200.1.1	1.135154528	NADH-dependent fumarate reductase, putative	Mitochondria [105]
**Ubiquitin metabolism**					
36	LdBPK_352500.1.1	1.239238063	ubiquitin-protein ligase, putative	Ubiquitin ligase [106]
37	LdBPK_160730.1.1	0.826720723	ubiquitin hydrolase, putative	protein deubiquitination (DUB10) [107]
38	LdBPK_351730.1.1	0.805438803	ubiquitin hydrolase, putative	protein deubiquitination (DUB6) [107]
** DNA transcription and cell cycle regulation**					
39	LdBPK_271511.1.1	-1.341701581	G histone H1 like	Binds to the entry/exit sites of DNA on the surface of the nucleosome core particle
40	LdBPK_010030.1.1	-1.265828128	Kinesin-13 1, putative	Only nuclear kinesin. Associates with the spindle during mitosis [35, 36]
41	LdBPK_323520.1.1	-1.124006202	CYC2-like cyclin, putative	CYC2 is a component of nuclear cyclin-dependent protein kinase holoenzyme complex and is important for promoting the transition from G1 to S-phase and for driving the cell cycle transition from G2 to mitosis as well [46, 47]
42	LdBPK_200030.1.1	-1.166528091	histone-lysine N-methyltransferase, H3 lysine-76 specific	Regulates gene transcription through the methylation of histone [108, 109]
43	LdBPK_010280.1.1	-0.902370628	pseudouridylate synthase-like protein	Pyrimidine nucleotide synthesis, biosynthesis, and salvage [110]
44	LdBPK_261320.1.1	-0.836546774	DNA ligase k alpha, putative	Unique DNA ligase localized to the kDNA disk [111]
45	LdBPK_241790.1.1	-0.802672206	cell division cycle protein 20	CDC20 is required for two microtubule-dependent processes, nuclear movement prior to anaphase and chromosome separation [45]
46	LdBPK_352090.1.1	1.287822313	kinesin, putative	Regulates microtubule dynamics [35]
** mRNA translation regulation**					
47	LdBPK_010790.1.1	-1.092328046	Eukaryotic initiation factor 4A1	Translation initiation [23, 24]
48	LdBPK_150320.1.1	-1.462798144	ribonucleoprotein p18, mitochondrial precursor, putative (fragment)	Binds to RNA during translational elongation [112]
49	LdBPK_010430.1.1	-1.004390562	ribosomal protein S7, putative (fragment)	Ribosomal subunits
50	LdBPK_010440.1.1	-0.903321576	ribosomal protein S7, putative	Ribosomal subunits
51	LdBPK_350630.1.1	1.021769303	Nup53/35/40-type RNA recognition motif-containing protein, putative	RNA binding protein

RNA-Seq data with log 2-fold change, cut off above 0.8 for upregulated and below -0.8 for down regulated genes, having known functions were listed. The transcript IDs were given according to *L*. *donovani* BPK282A1 strain as downloaded from the TriTrypDB (version 51) database (www.tritrypdb.org).

### Depletion of intracellular pool of LdPfn results in significantly reduced levels of eIF4A1 protein in LdPfn^+/-^ cells

**Fig 4:**
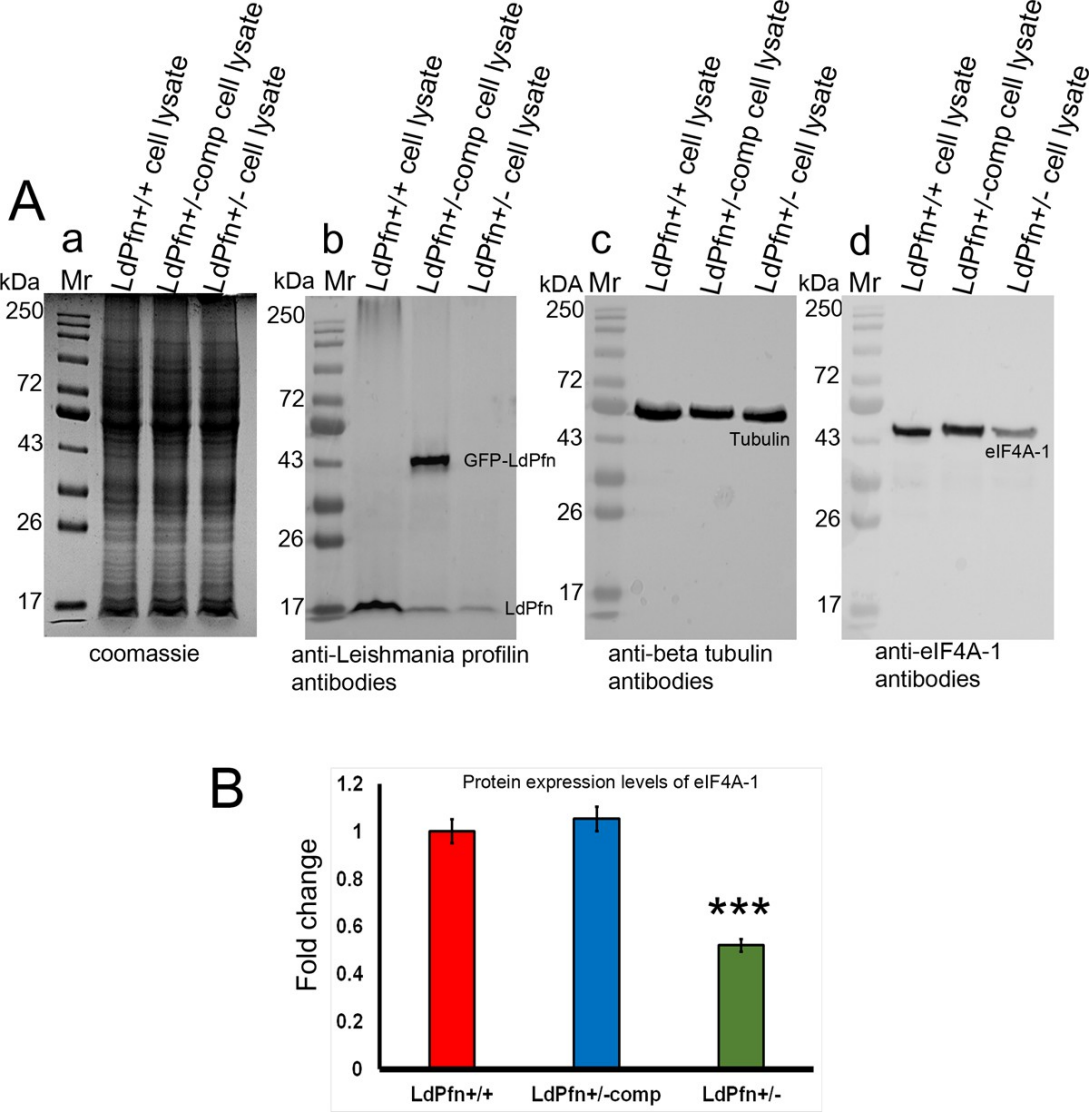
Western blot analysis showing depletion of eIF4A-1 protein in LdPfn^+/-^cells, compared to LdPfn^+/+^ and LdPfn^+/-comp^ cells. **(A)(a)** Coomassie blue-stained 12% SDS-polyacrylamide gel electrophoretogram showing equal loading of total cell lysates of LdPfn^+/+^, LdPfn^+/-comp^ and LdPfn^+/-^ cells. Mr, molecular weight markers; lane 1, LdPfn^+/+^ cell lysate; lane 2, LdPfn^+/-comp^ cell lysate; lane 3, LdPfn^+/-^ cell lysate. **(b)** Western blot of **‘a’** using anti-LdPfn antibodies. Mr, molecular weight markers; lane 1, LdPfn^+/+^ cells lysate showing expression of native profilin; lane 2, LdPfn^+/-comp^ cells lysate showing expression of both episomally expressed GFP-LdPfn (43kDa) and native LdPfn (16kDa); lane 3, LdPfn^+/-^ cells lysate showing depletion in the expression of native profilin. **(c)** Western blot of **‘a’** using anti-β-tubulin antibodies as loading control. Mr, molecular weight markers; lane 1, LdPfn^+/+^ cells lysate; lane 2, LdPfn^+/-comp^ cells lysate; lane 3, LdPfn^+/-^cells lysate. For generating LdPfn^+/-comp^ cells, the positive clone of GFP-LdPfn was transfected into LdPfn^+/-^ cells, as described earlier [11]. Hence, there are two bands, one for native LdPfn (16KDa) and other for GFP-LdPfn (43KDa), for profilin in LdPfn^+/-comp^ cells lysate immunoblot. **(d)** Western blot of **‘a’** using anti-eIF4A-1 antibodies. Mr, molecular weight markers; lane 1, LdPfn^+/+^ cells lysate; lane 2, LdPfn^+/-comp^ cells lysate; lane 3, LdPfn^+/-^ cells lysate, showing depletion in eIF4A-1 expression levels in LdPfn ^+/-^cells, compared to LdPfn^+/+^ and LdPfn^+/-comp^ cells. **(B)** The western blots of three independent experiments have been quantified using GelQuant.net software and the fold change in the protein expression levels of eIF4A1 was calculated by normalizing them against bands of β-tubulin. Around 50% reduction in protein expression levels of eIF4A1 was observed in LdPfn^+/-^ cells, compared to LdPfn^+/+^ and LdPfn^+/-comp^ cells.

The translation initiation factor 4A1 (eIF4A1), a DEAD-box RNA helicase, is a component of the translation initiation complex eIF4F, which binds to the cap structure of eukaryotic mRNA and helps in recruiting the small ribosomal subunit. As elF4A1 is a component of the LdPfn interactome (Table 1) and expression of its gene is significantly down-regulated in the LdPfn^+/-^ cells (Table 2), we examined whether the intracellular levels of elF4A1 protein have also been affected in these cells. For this, we probed the SDS- electrophoretograms of lysates of mid-log phase LdPfn^+/+^, LdPfn gene complemented LdPfn^+/-^ cells (LdPfn^+/-comp^) and LdPfn^+/-^ cells by western blotting, employing anti-LdPfn and anti-eIF4A1 antibodies (Fig 4A and S1 Fig). Results presented in Fig 4B clearly show that depletion of the intracellular pool of LdPfn results in about 50% reduction in cellular levels of eIF4A1 (Fig 4B) in *Leishmania* promastigotes. That the decreased expression of elF4A1 is caused due to depletion of LdPfn in *Leishmania* cells, was confirmed by episomal expression of GFP-LdPfn gene in the LdPfn^+/-^ cells (Fig 4A), which restored the elF4A1 levels to normal.

As cellular levels of key cell cycle proteins are strictly controlled by tightly regulated synthesis of such proteins during the G1-phase of the cell cycle [31–33], it may be inferred that the reduced expression of translation initiation factor 4A1 in LdPfn^+/-^ cells may adversely affect protein synthesis and consequently the cell division cycle in LdPfn^+/-^ cells. To confirm this conclusion, we analysed the cell division cycle in LdPfn^+/+^, LdPfn^+/-comp^ and LdPfn^+/-^ cells, using flowcytometry and immunofluorescence microscopy.

### LdPfn is involved in regulation of the G1-to- S phase progression

**Fig 5:**
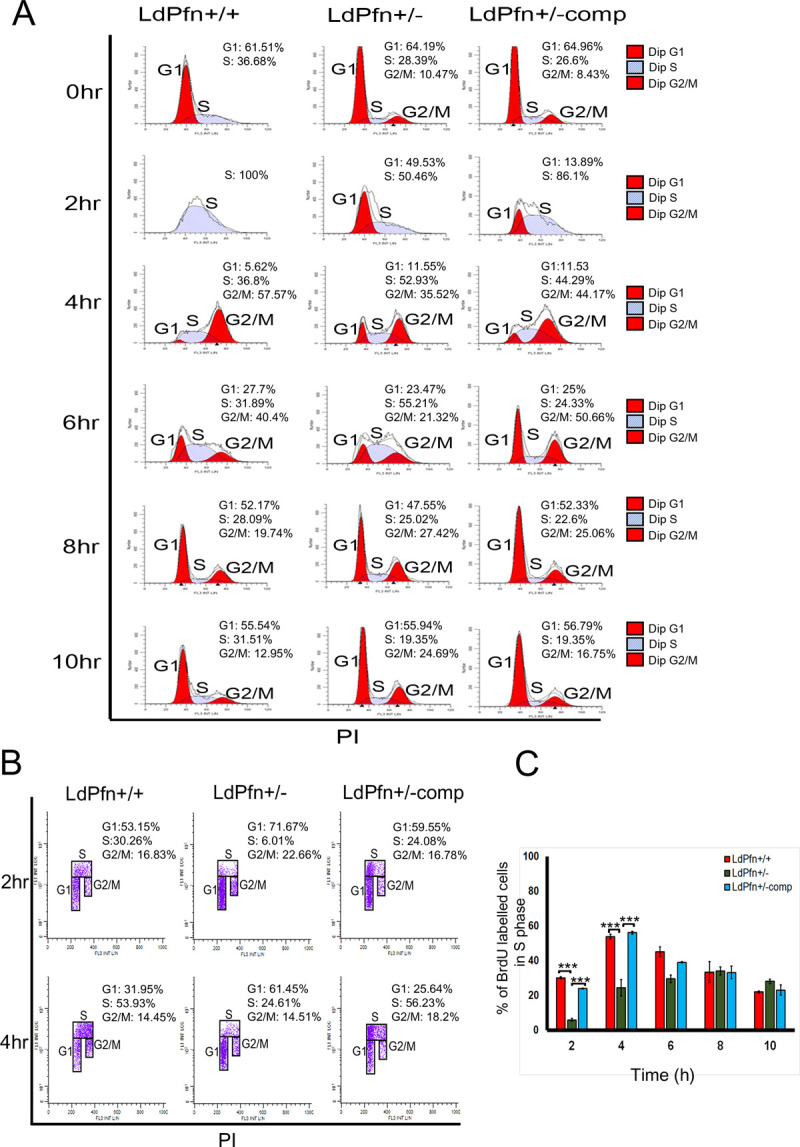
Retardation of cell cycle progression in LdPfn^+/-^ cells. **(A)** Representative flow cytometry data of LdPfn^+/+^, LdPfn^+/-^ and LdPfn^+/-comp^ cells. The samples were collected, after releasing hydroxyurea (HU) block, at 2 hours interval for up to 10 hours. 20,000 events were analysed at every time-point. Three independent experiments were performed, and one representative dataset is shown here. G1 (first red peak), S (grey peak) and G2/M (second red peak) phases are indicated in the histogram itself along with percent of cells in each phase. LdPfn^+/+^ cells entered into S-phase at 2 hours after release of HU block. However, transition of LdPfn^+/-^ cells from G1- to S-phase was considerably delayed, compared to LdPfn^+/+^ and LdPfn^+/-comp^ cells. **(B)** Representative flow cytometry data with BrdU incorporation in LdPfn^+/+^, LdPfn^+/-^ and LdPfn^+/-comp^ cells. The cells were collected, after releasing the HU block, at 2 hours interval for up to 10 hours, and then labelled with anti-BrdU antibodies, as described in ‘Materials and Methods’. 10,000 events were analysed at every time-point. Three independent experiments were performed, and one representative dataset of 2 hours and 4 hours is shown. G1, S and G2/M phases are indicated in the histogram along with the percent of cells in each phase. In LdPfn^+/-^ cells, a significantly lesser number of cells (6.01% and 24.61%, respectively, at 2 hours and 4 hours after releasing HU block) exhibited BrdU incorporation in S-phase, as compared to LdPfn^+/+^ cells (30.26% and 53.93%, respectively, at 2 hours and 4 hours after releasing HU block), and LdPfn^+/-comp^ cells (24.08% and 56.23%, respectively at 2 hours and 4 hours after releasing HU block). **(C)** Bar diagram showing considerably lesser number of BrdU labelled LdPfn^+/-^ cells (green bar) in S-phase at 2 hours and 4 hours after releasing the HU block, compared to LdPfn^+/+^ cells (red bar) and LdPfn^+/-comp^ cells (blue bar) at the same time points. p-value ***<0.001 at both 2 hours and 4hours after releasing the HU block.

As the gene expression in trypanosomatids is largely controlled at the post-transcriptional level, the main control points in *Leishmania* gene expression should therefore be mRNA degradation and translation [34]. This means that, the poor mRNA processing and translation would reflect poor gene expression in LdPfn^+/-^ cells. To test this possibility, we analysed the cell division cycle in LdPfn^+/-^, LdPfn^+/+^ and LdPfn^+/-comp^ cells. The cells were synchronized by treating them for about 12 hours with HU. Approximately 70% of the cells were synchronized at the G1/S border by HU treatment. After releasing the HU block, the cells were stained with PI to probe the total DNA content and then the samples were processed for the cell cycle analysis by flow cytometry. The LdPfn^+/+^ and LdPfn^+/-comp^ cells immediately entered the S-phase attaining the peak at 2 hours, and the G2/M peak was achieved at 4 hours after releasing the HU block. However, the LdPfn^+/-^ cells lagged in entering from the G1- to S-phase by at least 1 hour and remained mostly in the S-phase up to 6 hours (Fig 5A, S4A Fig). These results clearly revealed that profilin plays an important role in G1-to-S phase progression in *Leishmania* cell cycle. To further analyse this finding, we performed quantitative flow cytometric analysis with BrdU incorporation in LdPfn^+/+^, LdPfn^+/-^ and LdPfn^+/-comp^ cells to determine the number of cells present in different phases of cell cycle at different time points, after removal of the HU block. There were significantly higher number of LdPfn^+/-^ cells (71.7%), as compared to LdPfn^+/+^ (53.1%) or LdPfn^+/-comp^ cells (59.5%), in the G1-phase at 2 hours after releasing the HU block, whereas at the same time point, considerably lesser number of LdPfn^+/-^ cells (6.0%), compared to LdPfn^+/+^ (30.3%) or LdPfn^+/-comp^ (24.1%) cells, were present in the S- phase. Similarly, the number of LdPfn^+/-^ cells in the G1-phase was much higher (61.4%) than that of the LdPfn^+/+^ (31.9%) or LdPfn^+/-comp^ cells (25.6%) at 4 hours after release of the HU block, while only 24.6% LdPfn^+/-^ cells, compared to 53.9% LdPfn^+/+^ and 56.2% LdPfn^+/-comp^ cells, were in the S- phase at the same time period (Fig 5B and 5C).To rule out the possibility of toxic effects of HU on *Leishmania* promastigotes during synchronization conditions, we performed cytometric assay with BrdU incorporation in asynchronously growing mid-log phase *Leishmania* cells, without HU treatment, at 2 and 4 hours. In these conditions also, a significantly lesser number of LdPfn^+/-^ cells, compared to LdPfn^+/+^ and LdPfn^+/-comp^ cells, transited from the G1-to-S phase at both the time points (S4B and S4C Fig). These results demonstrate that LdPfn is involved in regulation of G1- to- S phase progression during *Leishmania* cell division cycle.

### LdPfn is involved in regulation of the mitotic spindle orientation

**Fig 6:**
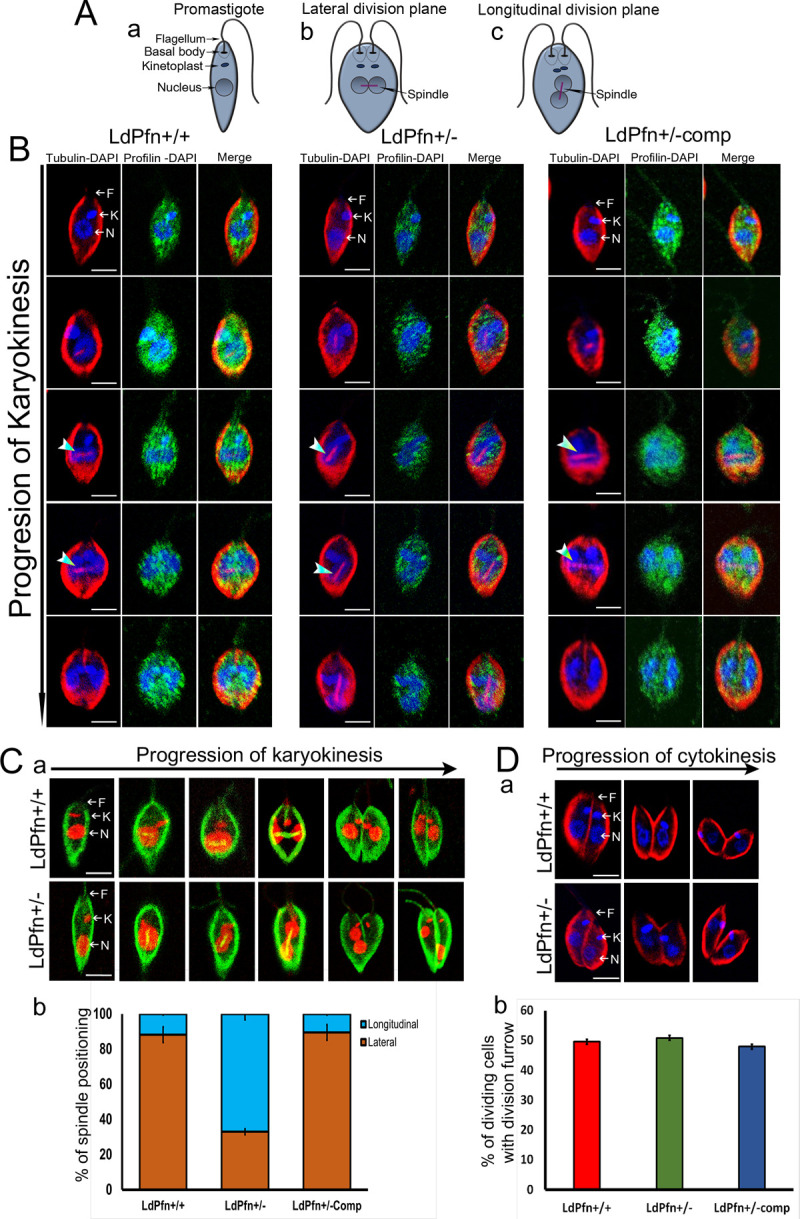
**(A)** Schematic representation of *L*.*donovani* **(a)** Promastigote **(b)** The plane of the nuclear division during karyokinesis when positioned parallel to the flagellar base in promastigote, lead to the formation of a laterally arranged spindle between the two dividing nuclei. **(c)** The plane of the nuclear division during karyokinesis when positioned perpendicular to the flagellar base in promastigote, lead to the formation of a longitudinally arranged spindle between the two dividing nuclei. **(B)** Representative immunofluorescence images of cell division pattern in LdPfn^+/+^, LdPfn^+/-^ and LdPfn^+/-comp^ cells. The cells were stained with anti-tubulin (red) and anti-LdPfn (green) antibodies and DAPI (blue). Analysis of dividing cells revealed that the plane of the nuclear division during karyokinesis in the LdPfn^+/+^ and LdPfn^+/-comp^ cells was positioned parallel to the flagellar base, leading to the formation of laterally arranged spindle between the two dividing nuclei. In contrast, in the LdPfn^+/-^ cells the dividing nuclei were arranged nearly perpendicular to the flagellar base, leading to a longitudinally formed mitotic spindle. Scale: 2μm; F, Flagellar base; K, Kinetoplast; N, Nucleus. **(C) (a)** The cells were alternately labelled with tubulin (green) and the nucleus and the kinetoplast with propidium iodide (red). In this case also, the nuclei division plane orientation in dividing LdPfn^+/-^ cells was altered, as compared to dividing LdPfn^+/+^ cells. Scale: 2μm. F, Flagellar base; K, Kinetoplast; N, Nucleus. **(b**) Quantification of the spindle positioning (lateral or longitudinal) in the dividing cells. Percentage of cells showing laterally positioned or longitudinally positioned mitotic spindle, as quantified after labelling the cells with tubulin. Significant number of LdPfn^+/-^ cells (n = 140) possessed nearly longitudinally positioned spindle, while LdPfn^+/+^ (n = 152) and LdPfn^+/- comp^ (n = 146) cells possessed laterally positioned spindle. p-value: 0.0002. **(D)** Representative fluorescence images of division furrow in LdPfn^+/+^ and LdPfn^+/-^cells. **(a)** The cells were labelled with tubulin (red) and the nucleus and kinetoplast with DAPI (blue) for analysis of cells undergoing cytokinesis. Scale: 2μm; F, Flagellar base; K, Kinetoplast; N, Nucleus. **(b)** No significant difference was observed in the percent of dividing cells with division furrow in LdPfn^+/-^ cells (n = 118), as compared to LdPfn^+/+^ (n = 125) and LdPfn^+/-comp^ (146) cells, indicating that the cytokinesis was not much affected by LdPfn depletion in *Leishmania* cells.

Kinesin 13–1 has been shown to be exclusively an intranuclear protein that regulates spindle assembly during mitosis, and its depletion leads to abnormalities in spindle structure and nuclear division [35, 36]. As shown in Table 2, depletion of LdPfn in *Leishmania* cells resulted in a significant down regulation of expression of the kinesin 13–1 transcript, we considered it of interest to investigate the segregation pattern of the nucleus during mitosis (Fig 6A). For this, the cells collected from the mitotic phase were labelled with anti-tubulin antibodies, anti-LdPfn antibodies, and DAPI or PI and then examined under the fluorescence microscope. Results revealed that the plane of the nuclear division during karyokinesis in the LdPfn^+/+^ cells was positioned parallel to the flagellar base, leading to the formation of a laterally arranged spindle between the two dividing nuclei. In contrast, in about 70% LdPfn^+/-^ cells, the dividing nuclei were arranged nearly perpendicular to the flagellar base, leading to a longitudinally formed mitotic spindle (Fig 6B). However, the division pattern in LdPfn^+/- comp^ cells was similar to that of the control LdPfn^+/+^ cells, indicating that the defect in the arrangement of dividing nuclei and orientation of spindle in LdPfn^+/-^ cells was a specific defect due to depletion in intracellular levels of profilin. To further confirm, we labelled the cells with ant-tubulin antibodies (green) and their nucleus and kinetoplast with PI (red) and then analysed them under a fluorescent microscope (Fig 6C). Quantification of the spindle positioning (lateral or longitudinal) in the dividing cells revealed that about 66% of LdPfn^+/-^ cells (n = 140) possessed nearly longitudinally positioned spindle, while only 11% of LdPfn^+/+^ cells (n = 152) and 10% of LdPfn^+/-comp^ cells (n = 146) showed such aberration (Fig 6C). These results demonstrate that profilin is involved in regulation of the mitotic spindle orientation and nucleus positioning during the mitotic phase in dividing *Leishmania* cells. However, quantification of the percent of dividing cells with division furrow (Fig 6D) showed no significant difference between the two cell types, suggesting cytokinesis has not been significantly affected.

## Discussion

Profilin is a ubiquitous actin-binding protein present in all eukaryotic cells, including unicellular eukaryotic organisms, such as *Plasmodium*, *Toxoplasma*, *Acanthamoeba Dictyostelium*, *Tetrahymena*, *etc*. It plays an important role in a number of cellular activities such as cell growth, intracellular trafficking, gene transcription, cell division, cell signalling, etc. [1–6]. Profilin has been suggested to be vital for the invasive blood stage *Plasmodium falciparum* and its complete deletion has been shown to have severe effects on viability of the parasites [37, 38]. In related parasite *Toxoplasma gondii*, this protein is known to be involved in cell motility, host cell invasion, immune evasion, and virulence [39]. Further, *Acanthamoeba castellanii* contains three profilin isoforms, each one of which exhibits different subcellular localization, diverse ligand binding properties and biological functions [40]. Furthermore, *Dictyostelium amoebae* lacking profilin isoforms I and II increase in cell size by up to 10 times, their motility is adversely affected, and a wide ring of filamentous actin accumulates beneath the plasma membrane, which blocks their development [41]. Besides this, in *Tetrahymena thermophila*, loss of profilin affected nuclear positioning, stomatogenesis, and cytokinesis [2]. Results of the present study clearly reveal that profilin is involved in regulation of G1-to-S phase progression and mitotic spindle orientation during *Leishmania* cell division cycle.

Cell division cycle may be visualised as a developmental process wherein a cell duplicates itself in to two progeny cells. During this process the cell first grows in size, replicates its chromosomes, segregates a full set of chromosomes to each of two new nuclei, and then divides into daughter cells [32]. In eukaryotes, the cell cycle comprises four discrete phases: G1, S, G2, and M. During the G_1_ phase, the cell congregates the building blocks of chromosomal DNA and the associated proteins, and also sufficient energy reserves for completing the replication process of each chromosome in the nucleus. Whereas in the S phase, DNA replication occurs to form identical pairs of DNA molecules. The building blocks of proteins and nucleic acids, namely amino acids and nucleobases/ nucleosides, are largely imported by the *Leishmania* parasite from the host environment through the plasma membrane by specific transporter proteins [42–44]. As depletion of LdPfn intracellular pool in *Leishmania* cells resulted in downregulation of genes that encode amino acids and nucleosides transporters, it may severely limit availability of the DNA and protein building blocks in G1 phase, which, in turn, may result in slowing down the progression of the G1- to-S phase transition. This is strongly supported by our quantitative flow cytometry data, which showed that the number of cells transited from G1-to-S phase at different time periods considerably decreased in LdPfn depleted *Leishmania* promastigotes, Furthermore, it is consistent with our observation that expression of genes that encode CDC20 and CYC2-like cyclin, which are known to play a critical role in regulation of trypanosomatids cell cycle [45–47], is markedly reduced in LdPfn^+/-^ cells.

Purines and pyrimidines are basic building blocks of nucleic acids. Distinct from their mammalian and insect hosts, *Leishmania* parasite lacks the metabolic machinery to produce purine nucleotides *de novo* and mainly relies on the acquisition of preformed purine nucleobases and nucleosides through its plasma membrane-bound specific permeases from the host [43]. The first such permease, designated LmaNT3, was identified in *L*. *major*, which showed about 33% identity to *L*. *donovani* nucleoside transporter 1.1 (LdNT1.1), and is, thus, a member of the equilibrative nucleoside transporter (ENT) family [44], which seems to be key elements controlling nucleoside and nucleotide pool for DNA synthesis [48]. As expression levels of the genes that encode nucleoside transporter 1 (LdBPK_151260.1.1; LdBPK_151250.1.1) was considerably reduced in LdPfn^+/-^ cells, it may be inferred that the decreased availability of purine nucleobases and nucleotides would significantly affect the DNA and RNA synthesis, and consequently the DNA replication, during the ‘S’ phase of the cell cycle.

DNA replication in *Leishmania* is primarily determined by the active transcription [49]. In this group of organisms RNA polymerase II transcription is polycistronic and individual mRNA are excised by trans-splicing and polyadenylation [34]. The lack of individual gene transcription control is mainly compensated by post-transcriptional mechanisms, including tight translational control and regulation of mRNA stability/translatability by RNA-binding proteins [50]. As LdPfn interacts with the proteins that are involved in mRNA processing and translation initiation, these interactions would be adversely affected in LdPfn depleted cells, which in turn could affect the mRNA processing and translatability.

Translation of mRNA into proteins is initiated with the binding of the multimeric translation initiation complex elF4F to the cap structure of mRNA present at its 5’ end. The elF4F complex is comprised of the cap binding protein, elF4E, the highly conserved mRNA helicase, elF4A, and the large scaffolding protein having binding sites for both elF4E and elF4A, elF4G is responsible for recruiting ribosomal subunits to the initiation codon of mRNA. Although several potential elF4F homologues have been identified in the *L*. *major* database, only three elF4E, two elF4A and one elF4G (elF4G3) have so far been characterized [23]. While elF4A1 and elF4E3 have been reported to be abundantly present, the other proteins are either moderately abundant or not detected in *L*. *major* promastigotes [23]. Similar *to L*. *major*, elF4A-1 is very abundant and predominantly cytoplasmic protein also in *T*. *brucei*, and its depletion to 10% of regular levels dramatically decreases protein synthesis one cell cycle following double-stranded RNA induction and blocks cell proliferation [24]. Based on these findings, it has been suggested that only the elF4A1 protein is involved in protein synthesis [24]. As the expression of elF4A1 was found to depend on the intracellular levels of LdPfn, the decreased availability of elF4A1 in LdPfn^+/-^ cells should affect the protein translation process.

The direction in which a cell divides is determined by the orientation of its mitotic spindle at metaphase. The spindle orientation in eukaryotes is controlled by a conserved biological machine that mediates a pulling force on astral microtubules. Constraining the localization of this machine to only certain regions of the cortex can thus determine the orientation of the mitotic spindle [51]. Kinesins and dyneins are microtubules-dependent motor proteins that transport cargo in opposite directions along microtubules. However, the kinesin 13 family of proteins in trypanosomatids do not possess cargo transporting property, but they do depolymerize microtubules at their ends, and thereby control microtubule length [36]. It has been shown that in kinesin 13 family of proteins, kinesin 13–1 is exclusively intranuclear in both *T*. *brucei* and *L*. *major*, where it mainly localizes to the mitotic spindle and spindle poles and plays a central role in regulating the spindle assembly during mitosis [35, 36]. As expression of the gene that encodes kinesin 13–1 was significantly down regulated in the LdPfn depleted *Leishmania* cells, we speculate that LdPfn might have been involved in regulating the spindle orientation by controlling the expression of kinesin 13–1 protein in these cells.

Based on our proteomic and transcriptomic data analyses, it appears that besides having a role in cell cycle regulation, LdPfn may also be involved in regulating the *Leishmania* mitochondrial activity. *Leishmania* has a single large mitochondrion, which is distributed in branches under the subpellicular microtubules and in a specialized region, called kinetoplast, it houses its unusual genome, known as kDNA [52]. Mitochondria in eukaryotic cells are responsible for oxidative phosphorylation, and harness energy from numerous substrates through electron transport chains. They are semi-autonomous cell organelles which have their own DNA and protein synthesizing machinery [52, 53]. However, *Leishmania* mitochondrion is completely devoid of tRNA-encoding genes and hence, it imports nucleus-encoded tRNAs for protein synthesis [54] through a receptor-mediated pathway [55, 56]. Several mitochondrial proteins that are involved in the electron transfer mechanisms and also the outer membrane protein, porin, which is the main metabolite channel and is required to support efficient oxidative phosphorylation in *T*. *brucei* [22], interact with LdPfn in *Leishmania* promastigotes, it may be envisaged that activity of these proteins would be adversely affected by depleting intracellular pool of LdPfn. This is consistent with our finding that expression of transcripts that encode for proteins involved in mitochondrial electron transport activities, mitochondrial protein folding, and protein import across the mitochondrial outer membrane was significantly decreased in LdPfn^+/-^ cells. Further, protein import in trypanosomatid mitochondrion is mediated by the archaic translocase of the outer membrane (ATOM) complex, consisting of six subunits [57, 58]. Among them, expression of transcripts encoding ATOM14 and ATOM11 was significantly reduced in the LdPfn^+/-^ cells. As knockdown of ATOM14 in *T*. *brucei* has been shown to result in growth arrest and drastically reduced protein and tRNA import into the mitochondrion [59] and ATOM11, which is exclusively present in trypanosomatids and is known to have a direct role in mitochondrial tRNA import [60], These results strongly suggest that LdPfn could play an important role in regulating the protein synthesis and oxidative phosphorylation in *Leishmania* mitochondrion.

Finally, our earlier studies have shown that LdPfn binds actin, PLP motif-containing proteins and membrane phosphoinositides, especially PI(3,5)P2, PI(4,5)P2 and PI(3,4,5)P3 [11]. As membrane phosphoinositides play a central role in regulation of cell signalling, membrane trafficking, actin remodelling, nuclear events and intracellular transport [61–63], depletion of LdPfn intracellular levels should affect LdPfn interactions with these lipids, which in turn should affect phosphoinositide metabolism and consequently their cellular functions, including regulation of actin dynamics, cellular metabolism and membrane signalling. This is well supported by our present and earlier findings that depletion of LdPfn in *Leishmania* cells not only affected the intracellular transport [11], but it appeared to affect also the expression of proteins that regulate cell signalling, membrane transport, and actin remodelling. It has earlier been suggested that cortical actin remodelling plays a key role during cytokinesis and early mitosis in eukaryotic cells [64]. However, in *Leishmania* cells, the role of actin dynamics has been shown only in the basal body and kinetoplast separation, cleavage furrow progression and flagellar pocket division [65]. Although no such aberrations were observed during cytokinesis of the LdPfn depleted cells, based on the presently available data, we cannot completely rule out the role of actin remodelling in cell division cycle of these cells.

## Supporting information

S1 Table Primer list used for performing quantitative real-time PCR. (XLSX)

S2 Table Potential binding partners of *L*.*donovani* profilin. Four proteins were identified as present in all three GST-profilin pulldown assays, while 34 proteins were identified in at least two GST-profilin pulldown assays. Only proteins that were not detected in any of the GST-alone pulldown controls were considered. The protein IDs were given according to *L*. *donovani* BPK282A1 strain as downloaded from the TriTrypDB (version 51) database (www.tritrypdb.org).
(XLSX)

S3 Table Statistics for RNA-Seq data sets. Reads alignment was done by Bowtie2, using the *L*. *donovani* genome (*Leishmania donovani* BPK282A1, NCBI taxon ID:981087) [17].
(XLSX)

S4 Table Transcripts of differentially expressed genes (DEGs) upregulated DEGs and downregulated DEGs with unknown functions, with p-value<0.05. The transcript IDs were given according to *L*. *donovani* BPK282A1 stain as downloaded from the TriTrypDB (version 51) database (www.tritrypdb.org). Hypothetical protein: Conserved: predicted protein of unknown function that is also annotated in other trypanosomatid genomes (GeneDB.org). Hypothetical protein: predicted bioinformatically. Hypothetical protein unknown function: non-demonstrated protein-coding function.
(XLSX)

S1 Fig The original, uncropped and unadjusted images underlying all blots and gels. (PDF)

S2 Fig Overview of the experimental design. The flowchart represents RNA-seq workflow and bioinformatics analysis workflow.
(TIF)

S3 Fig Principal Component Analysis (PCA) scatter plot. PCA scatter plot of differentially expressed genes (DEGs).
(TIF)

S4 Fig Cell cycle progression. **(A**) Graphical representation of the percentage of LdPfn^+/+^, LdPfn^+/-^ and LdPfn^+/-Comp^ cells in different stages in the cell cycle, G1-phase (green colour, diamond), S-phase (red colour, square) and G2/M phase (blue colour, triangle) at 2 hours interval up to 10 hours, after removing the hydroxyurea (HU) block. LdPfn^+/+^ and LdPfn^+/-comp^ cells reached S-phase in 2 hours, but LdPfn^+/-^ cells showed slow progression through S-phase for up to 6 hours after release of the HU block. **(B)** Representative flow cytometry data with BrdU incorporation in mid-log phase unsynchronized LdPfn^+/+^, LdPfn^+/-^ and LdPfn^+/-comp^ cells. The cells were collected at 2 hours and 4 hours. 10,000 events were analysed at every time-point. Three independent experiments were performed, and one representative dataset is shown here. G1, S and G2/M phases were indicated in the histogram along with the percent of cells in each phase. In LdPfn^+/-^ cells, a significantly lesser number of cells exhibited BrdU incorporation in the S-phase, compared to LdPfn^+/+^ cells and LdPfn^+/-comp^ cells at both the time points. **(C)** Bar diagram showing BrdU incorporation in mid-log phase unsynchronized cells, without HU treatment, at 2 and 4 hours. A significantly reduced number of BrdU labelled LdPfn^+/-^ cells (green bar) entered the S-phase, compared to LdPfn^+/+^ cells (red bar) and LdPfn^+/-comp^ cells (blue bar) at 2 hours and 4 hours. p- value: ***<0.001 at 2hours, *<0.05 and **<0.01 at 4 hours.
(TIF)
