# Supplementary figures and images for: Profilin is involved in G1 to S phase progression and mitotic spindle orientation during Leishmania donovani cell division cycle
Source: PLoS One. 2022 Mar 22;17(3):e0265692. doi: 10.1371/journal.pone.0265692 (PMC8939790; doi:10.1371/journal.pone.0265692)

Raw images of Fig 1C

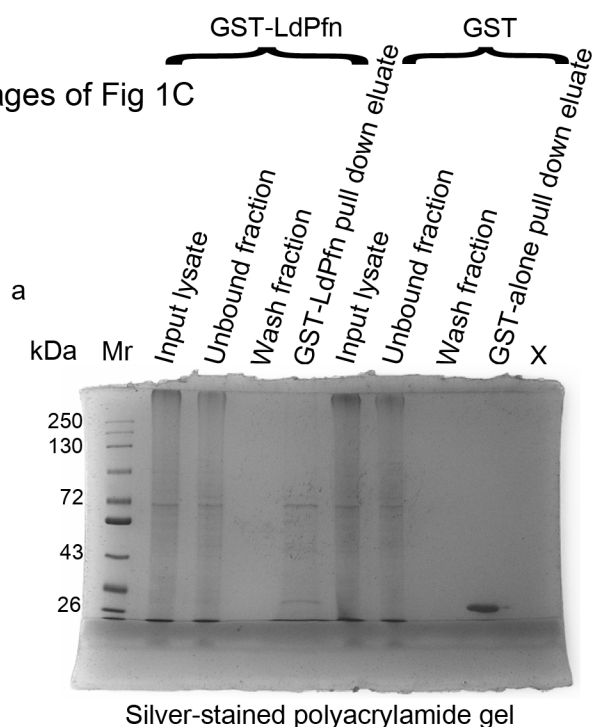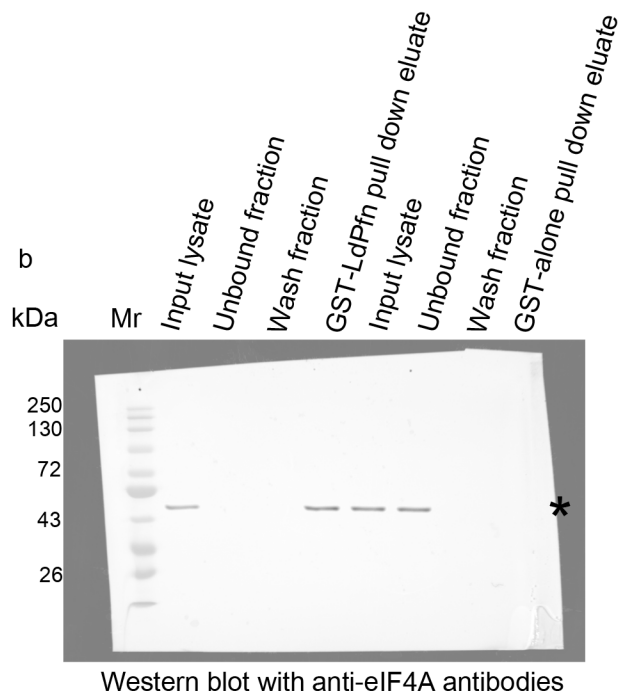

Raw images of Fig 4A

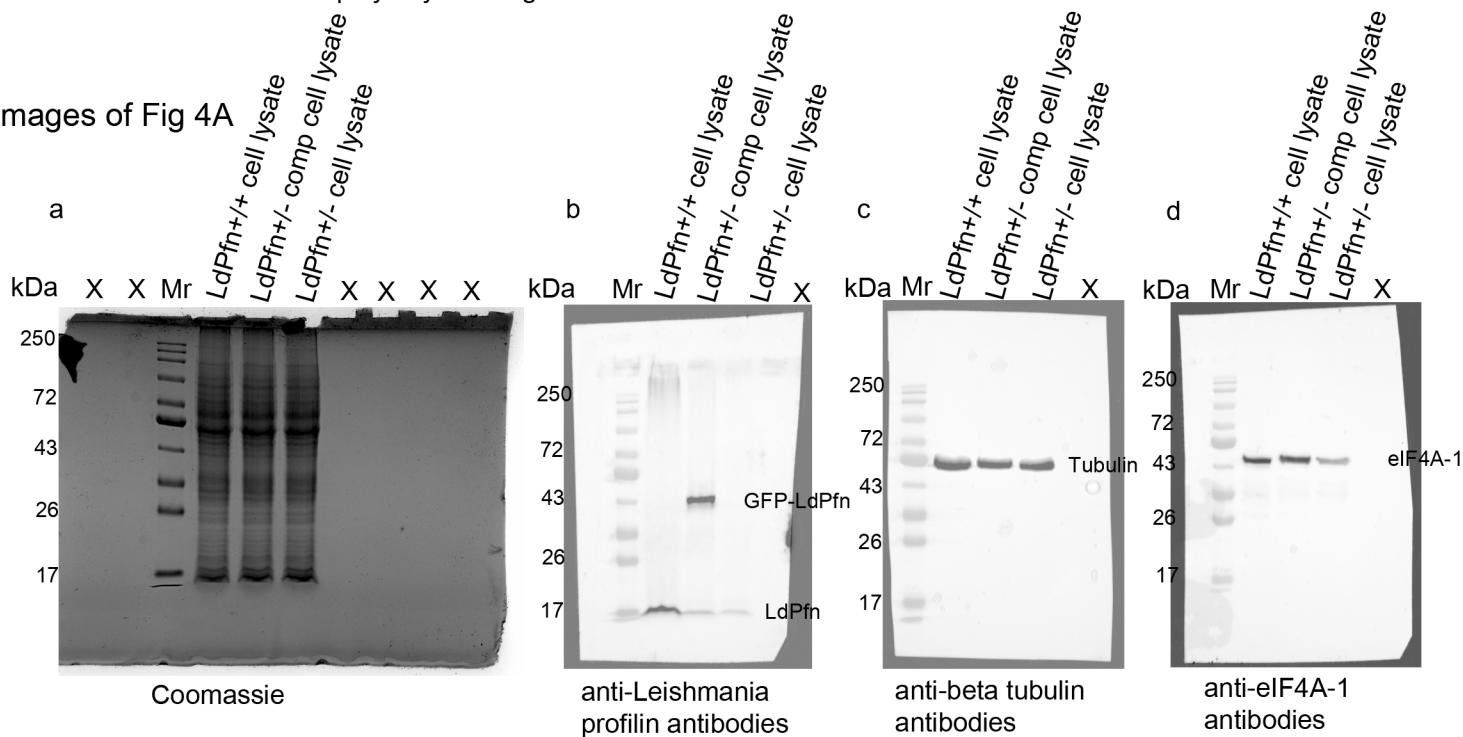

Supplement: S1 Fig — (PDF) [file pone.0265692.s005.pdf]

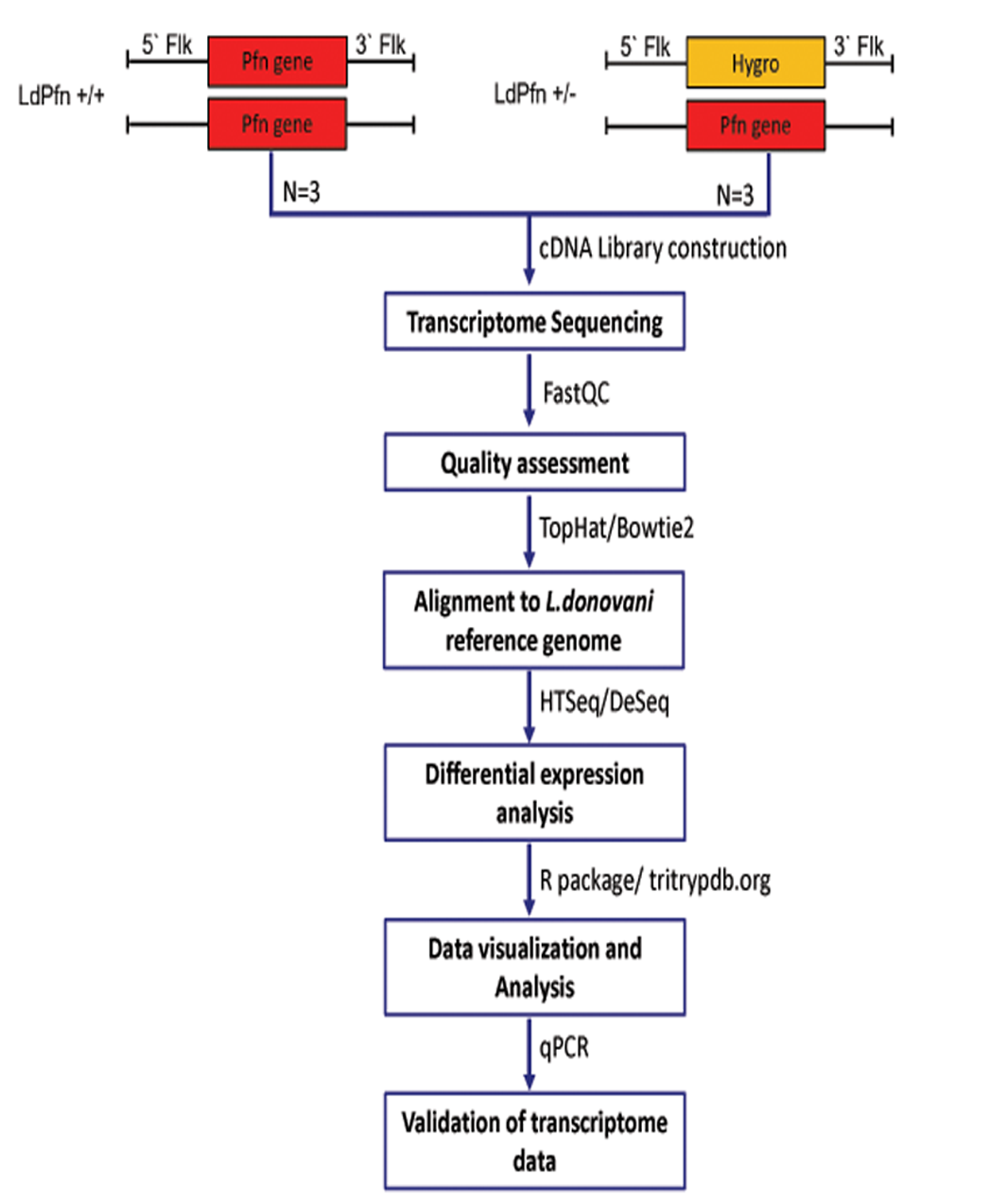

Supplement: S2 Fig — The flowchart represents RNA-seq workflow and bioinformatics analysis workflow. (TIF) [file pone.0265692.s006.tif]

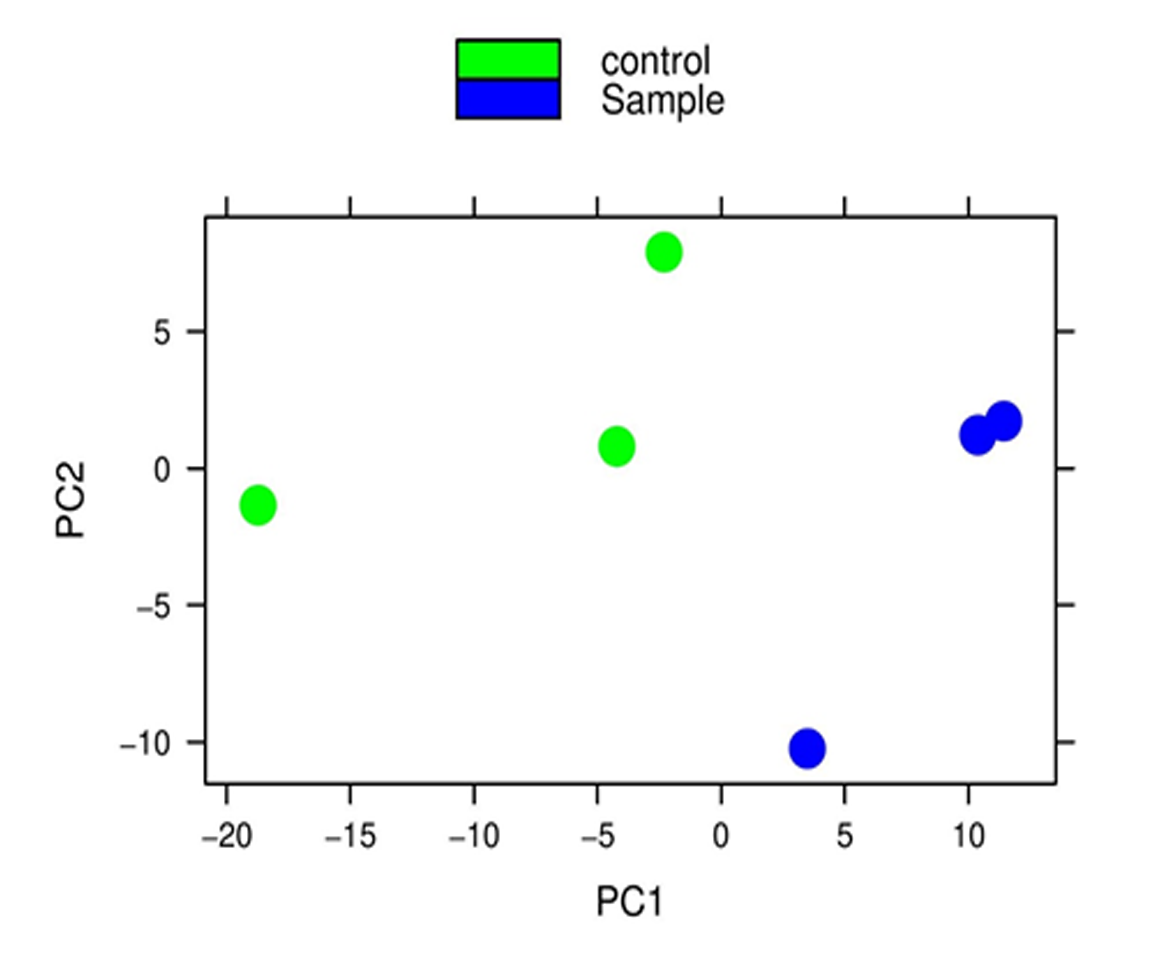

Supplement: S3 Fig — PCA scatter plot of differentially expressed genes (DEGs). (TIF) [file pone.0265692.s007.tif]

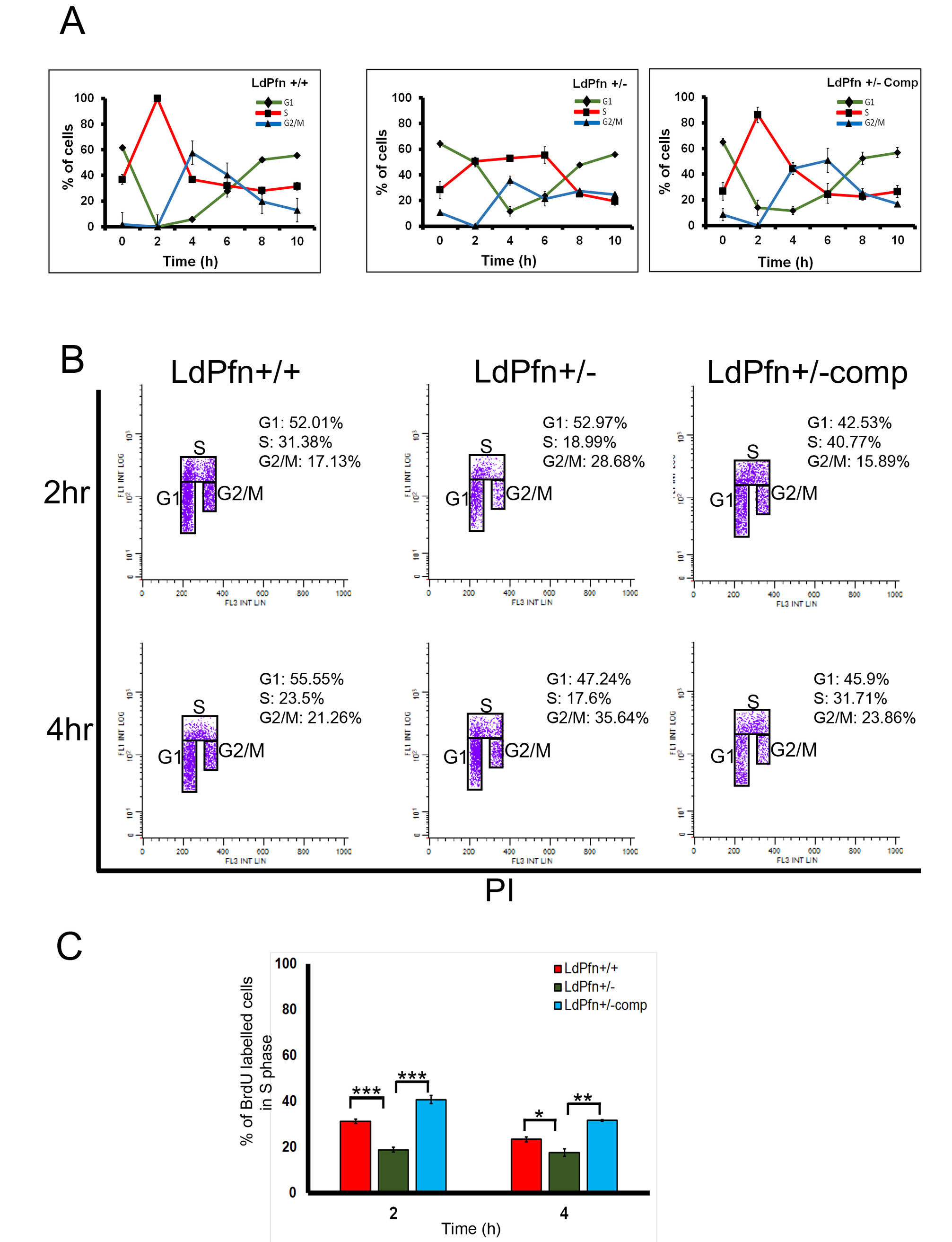

Supplement: S4 Fig — (A) Graphical representation of the percentage of LdPfn+/+, LdPfn+/- and LdPfn+/-Comp cells in different stages in the cell cycle, G1-phase (green colour, diamond), S-phase (red colour, square) and G2/M phase (blue colour, triangle) at 2 hours interval up to 10 hours, after removing the hydroxyurea (HU) block. LdPfn+/+ and LdPfn+/-comp cells reached S-phase in 2 hours, but LdPfn+/- cells showed slow progression through S-phase for up to 6 hours after release of the HU block. (B) Representative flow cytometry data with BrdU incorporation in mid-log phase unsynchronized LdPfn+/+, LdPfn+/- and LdPfn+/-comp cells. The cells were collected at 2 hours and 4 hours. 10,000 events were analysed at every time-point. Three independent experiments were performed, and one representative dataset is shown here. G1, S and G2/M phases were indicated in the histogram along with the percent of cells in each phase. In LdPfn+/- cells, a significantly lesser number of cells exhibited BrdU incorporation in the S-phase, compared to LdPfn+/+ cells and LdPfn+/-comp cells at both the time points. (C) Bar diagram showing BrdU incorporation in mid-log phase unsynchronized cells, without HU treatment, at 2 and 4 hours. A significantly reduced number of BrdU labelled LdPfn+/- cells (green bar) entered the S-phase, compared to LdPfn+/+ cells (red bar) and LdPfn+/-comp cells (blue bar) at 2 hours and 4 hours. p- value: ***<0.001 at 2hours, *<0.05 and **<0.01 at 4 hours. (TIF) [file pone.0265692.s008.tif]
